# Intranasal drug delivery: opportunities and toxicologic challenges during drug development

**DOI:** 10.1007/s13346-020-00891-5

**Published:** 2021-01-25

**Authors:** Lea-Adriana Keller, Olivia Merkel, Andreas Popp

**Affiliations:** 1 Preclinical Safety, AbbVie Deutschland GmbH & Co. KG, Knollstrasse, 67061 Ludwigshafen, Germany; 2 Department of Pharmacy, Pharmaceutical Technology and Biopharmacy, Ludwig-Maximilians-University, Butenandtstraße 5-13, 81337 Munich, Germany

**Keywords:** Intranasal drug delivery, Olfactory pathways, Nose-to-brain-delivery, Nasal toxicity

## Abstract

**Abstract:**

Over the past 10 years, the interest in intranasal drug delivery in pharmaceutical R&D has increased. This review article summarises information on intranasal administration for local and systemic delivery, as well as for CNS indications. Nasal delivery offers many advantages over standard systemic delivery systems, such as its non-invasive character, a fast onset of action and in many cases reduced side effects due to a more targeted delivery. There are still formulation limitations and toxicological aspects to be optimised. Intranasal drug delivery in the field of drug development is an interesting delivery route for the treatment of neurological disorders. Systemic approaches often fail to efficiently supply the CNS with drugs. This review paper describes the anatomical, histological and physiological basis and summarises currently approved drugs for administration via intranasal delivery. Further, the review focuses on toxicological considerations of intranasally applied compounds and discusses formulation aspects that need to be considered for drug development.

**Graphical abstract:**

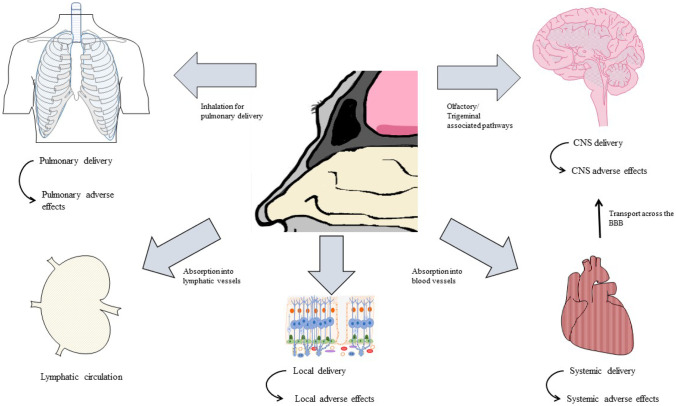

## Introduction

Nasal administration has been used for therapeutic reasons for centuries. As the respiratory tract is a primary contact zone for the environment, it represents a gateway, not only for infectious particles such as bacteria and viruses but also for potential treatments. In the past century, the use of intranasally (IN) administered drugs was mainly restricted to treating topical symptoms of seasonal rhinitis or infectious diseases of the respiratory tract, for example. At the end of the twentieth century, the nasal delivery route became more prominent as an alternative route to treat systemic symptoms such as in cardiovascular indications. The possibility to deliver drugs to the central nervous system (CNS) through nasal pathways remained unexplored until in 1991. William Frey II proposed a patent for a nasal drug delivery method to treat neurological disorders in the brain [1]. Subsequently, there was an increased interest in nasal delivery especially for the growing field of nose-to-brain-delivery (ntb). Ntb delivery, in contrast to systemic delivery, presents a promising alternative enabling the delivery of therapeutic drugs to the CNS, while bypassing the blood-brain barrier (BBB). Compared with conventional drug delivery approaches, ntb delivery represents a non-invasive method, to access the CNS directly through the olfactory or trigeminal nerves. Each drug formulation favours different transport mechanisms, including intracellular and extracellular pathways, from the nasal cavity to the higher brain regions. During drug formulation development, the influence of absorption into the blood circulation, lymphatic systems and into the cerebrospinal fluids has to be considered. For several CNS disorders, specific and efficient therapeutic proteins already exist, and for others, new biologics need to be developed. What they have in common is their potential to improve therapy outcomes and to reduce side effects compared with currently approved systemic medications or compared with the systemic doses currently necessary if delivery to their site of action is enhanced. While there are a number of approved drug formulations for local and systemic indications, the development of nasal drug formulations for CNS delivery is still a challenge. This review paper not only discusses the possibilities and advantages of intranasal drug delivery (INDD) but also highlights its current limitations and toxicological considerations. Anatomical, histological, physiological and pathological information about the nose, together with data on drugs and their formulations, was gathered, to further discuss their influence on INDD and drug development. The future perspectives for the establishment of intranasal drugs for local, systemic and CNS indications are highlighted in this review paper.

## Anatomy of the nose

### The nasal anatomy

**Fig. 1:**
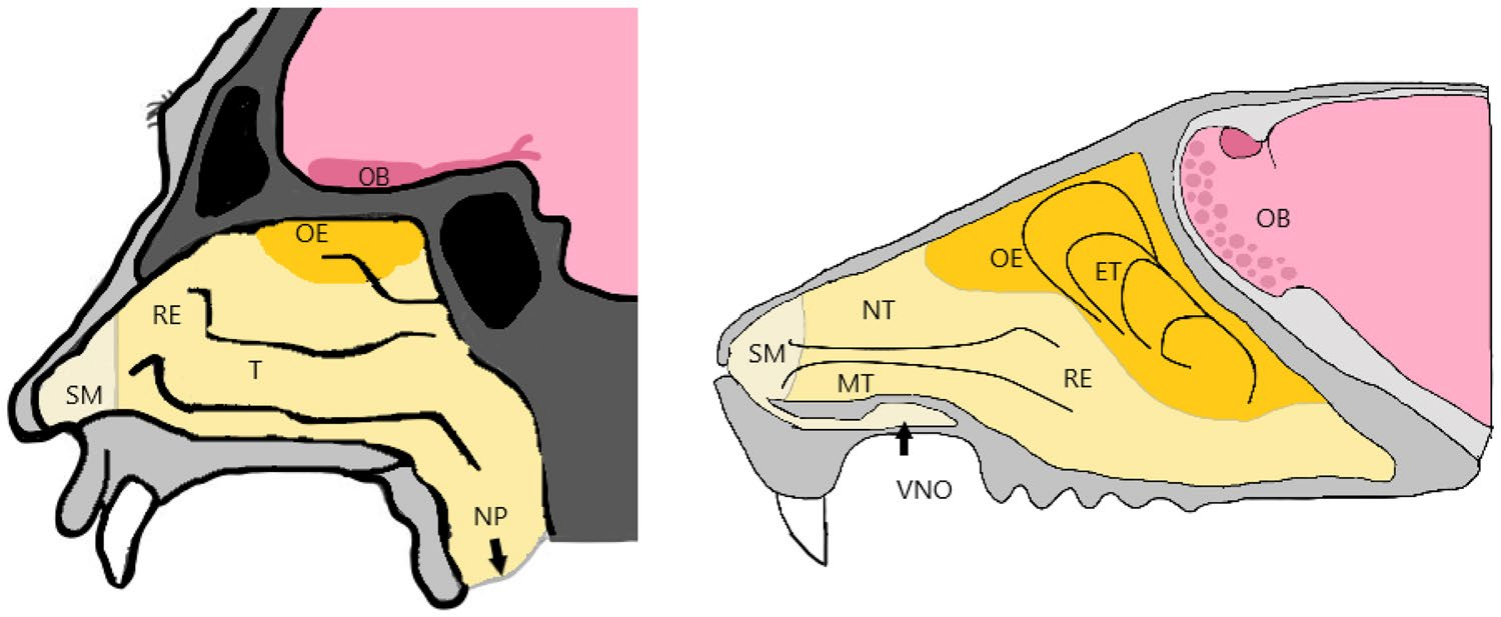
Nasal cavity of humans (left) and rodents (right), showing the different regions within the nose. Starting with the squamous mucosa (SM) right at the nostril openings. The respiratory epithelium (RE) covers the main part of the nasal cavity in humans. Humans have three turbinates (T), the inferior turbinate, the middle turbinate as a part of the RE and the superior turbinate in the olfactory epithelium (OE). Rodents have the maxilloturbinates and nasoturbinates in the RE and the ethmoturbinates in the OE. The olfactory bulb (OB) is in close proximity to the cribriform plate and connected to the OE via the axons of the sensory neurons projecting towards the brain. The rodent nasal cavity further has a predominant vomeronasal organ (VNO) which also takes part in the olfaction of specific compounds

The nose is a structurally and functionally complex organ and hosts one of the 5 main senses. Besides filtration, humidification and temperature control, the olfaction is an important function of the nose, not only for animals to detect food, predators and mates, but also for humans [2, 3]. The principal structure of the nose is in general comparable between rodents, which are commonly used as laboratory animals, and humans. The nasal cavity is divided into two areas which reach from the nostrils towards the nasopharynx. They can be separated into three regions: the vestibular region, the respiratory region and the olfactory region. The vestibular region in humans spans over ~ 0,6 cm^2^ and is located at the nostril opening. This area contains nasal hairs, squamous epithelial cells and few if any ciliated cells. The respiratory area covers with 150 cm^2^ the lateral part of the nose and represents the largest area. In humans, the respiratory region makes up to 80–90%, while in rodents, it only covers 50% of the nasal cavity (Fig. 1). The respiratory region is a pseudostratified and columnar epithelium and is the most vascular region. It consists of mainly four cell types: goblet cells which secrete mucin for the mucus layer, ciliated, non-ciliated columnar and basal cells. The third region is the olfactory region which in humans covers ~10 cm^2^ of the nasal cavity [3–6]. In humans, the nasal cavity is connected to the paranasal sinuses. The paranasal sinuses are four paired spaces named after the bone in which they are located (frontal sinus, sphenoid sinus, ethmoid air cells and maxillary sinus). Even though the function of the paranasal sinuses is unclear, they support functions such as reducing head-weight, cleaning and humidifying inhaled air and improving the resonance of sound and speech [7, 8]. Besides differences in the surface area of the olfactory epithelium between preclinical animal models and humans, there are also other translational limitations, which should be considered. The accessibility of the olfactory epithelium of rodents and humans, for example, is different, as the nasal cavity of rodents is narrower and thus less accessible than in humans [9, 10]. In humans, the collection of olfactory mucosa can be carried out by an ear, nose and throat surgeon using a local anaesthetic, while in rats, the animal has to be euthanised before removing the nasal bone to access to the olfactory epithelium [10]. More recently, new surgical methods for olfactory epithelium biopsies in rats without euthanasia have been described [9]. As shown in Fig. 1, other anatomical factors such as the bend from the nostrils into the nasal cavity, the length and volume, the structure of the conchae and the presence of a septal window can cause differences in nasal uptake and absorption between species. Further physiological conditions of the nose, dosage form factors, administration and sampling techniques as well as choice of administration device should be considered during species selection, as they can limit the translation towards the human situation [11–13].

### The olfactory region 

**Fig. 2:**
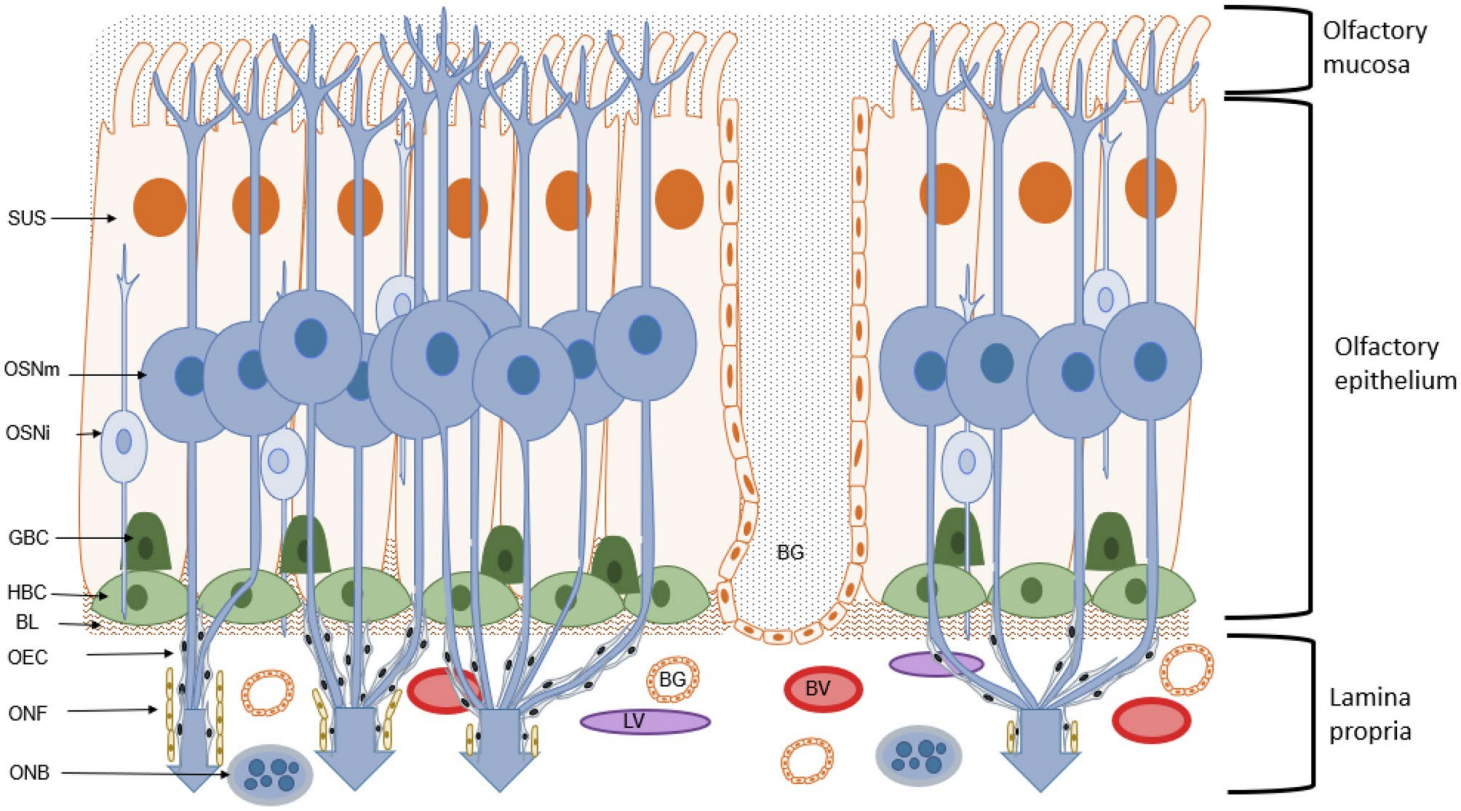
Structure and composition of the olfactory mucosa. In the posterior part of the nasal cavity, the olfactory mucosa, together with the olfactory epithelium (OE) and the lamina propria (LP), represents the first contact zone of environmental cues towards the human body. Within the OE, the mature olfactory sensory neurons (OSNm) are projecting their axons towards the olfactory bulb (OB), where they form glomeruli with the dendrites of mitral cells. The axons of OSNms are enclosed by olfactory ensheating cells (OECs) and olfactory nerve fibroblasts (ONFs). The axons together with the OEC and ONF form the olfactory nerve bundles (ONBs) in the lamina propria. The OE further consists of sustentacular (SUS) and mucus producing Bowman’s glands (BGs). In the middle part of the OE are the immature ORN (ORNi). The OE is surrounded by a layer of immature basal cells, the globose (GBC) and horizontal basal cells (HBCs). The lamina propria (LP) is seperated from the OE by a basal lamina (BL). Further, the LP also contains blood vessels (BVs) and lymphatic vessels (LVs)

The olfactory epithelium (OE) is the first contact zone of environmental information to the body, situated in the upper part of the nasal cavity, called the olfactory cleft. While in humans this region is only ~10% of the area, in rodents which are mainly used for IN administration studies, the olfactory region can make up to 50% of the total area [6, 14, 15]. It contains several cell types including tubular Bowman’s glands which secrete a mucus layer that covers the epithelium. Horizontal basal cells (HBCs) and globose basal cells (GBCs) are located close to the lamina propria and act as progenitor cells for the other cell types. Further, the OE encloses olfactory sensory neurons (OSNs), also called olfactory receptor neurons (ORNs), which are surrounded by supporting cells (Fig. 2). The OSNs are unmyelinated, bipolar cells, having a dendritic extension to the mucosal surface, where cilia carry olfactory receptors (ORs) [16]. From the cell body of OSN, an axon reaches through the cribriform plate of the ethmoid bone (which separates the nasal cavity from the brain) directly to the olfactory bulb (OB). The axons are enclosed by interconnecting olfactory ensheating cells, which are additionally covered by layers of neural fibroblasts (ONFs). The ONF layers form the perineural sheath. Together with the OEC, the ONF encompass the OSN axons along the olfactory nerve until they reach the OB [17].

The specialised ORs represent the first part in the signalling pathway of the olfactory system. They possess the enormous discrimination power of this system through which humans can distinguish between thousands of different odours. As they belong to the large gene family of the G-protein coupled receptors (GPCRs), odorant receptors (ORs) contain the GPCR characteristic seven-transmembrane domain (7TMD) structure [18]. The specificity of the ORs towards molecules is based on their extensive sequence diversity within the transmembrane domains, where the ligand binds. The consensus view is that one OSN expresses one OR gene, but this rule may not be conclusive [19, 20]. Nevertheless, the observations revealed a mechanism of receptor gene choice, in which a cell selects one allele but can switch at low frequencies, as long as a functional receptor is expressed [21, 22]. Each OSN extends a single dendrite to the surface of the epithelium. From there cilia, which are enriched with OR extend to contact odorants in the air. The OR interacts with its ligand, which results in the activation of heterotrimeric G-proteins, G-protein α subunit (Gαolf) and G beta-gamma complex (Gβγ). The activated Gαolf then activates type III adenylyl cyclase, which catalyses the production of cyclic AMP (cAMP) from ATP. The increase of the cAMP-level opens the cyclic nucleotide-gated (CNG) channel, which leads to an influx of sodium and calcium ions, and hence to the depolarisation of the neuron. The initial depolarisation is amplified through the opening of other channels and chloride efflux [23]. The OR interacts with its ligand and the signal is transmitted along the axons towards the OB. The OSNs which express the same OR, all send their axons to a distinct area, called glomerulus, in the OB [23]. Thus, the identity of the OSN through the expression of a specific OR seems to play a role for the axonal wiring to the OB.

### The axonal wiring towards the olfactory bulb

The first site for processing the olfactory information is the OB, a forebrain structure, where the axons of each olfactory sensory neuron with the same identity meet. For a long time, the question on how the axonal connection of 1000 different types of odorant receptors is organised, in such a precise way, kept the field of olfactory research busy [24].

The mammalian OB has a simple cortical structure with thousands of signal-processing modules called “glomeruli.” Those glomeruli represent the olfactory sensory convergence centre, where all inputs of odorant receptors, belonging to the same type, transmit their either signals to mitral or tufted cells. After the first axonal wiring to the mitral cell, which is processed by the local neuronal circuits, that mediate synaptic interactions, the information is sent to the olfactory cortex [25]. The glomeruli of the OB represent a map of the axons of OSNs and therefore their OR. Hence, studies have shown that each odour has a specific characteristic pattern of glomerular activity [25, 26]. This relationship of odorant receptor pattern in OB and OE is called zone-to-zone projection. The OB has four zones (zone I, II, III, IV) lining from dorsomedial to ventrolateral parts of the OE. One striking finding was that glomeruli which are located next to each other respond to molecules with similar structures [24, 25]. It is now clear that the zones overlap and do not have a sharp boarder to each other [27].

Another aspect is how this map is maintained and how OSN axons target and converge in the appropriate region of the OB. Many studies have been carried out to investigate the underlying hierarchy of cues, but the axonal wiring is a complex process which cannot be explained by simple single gradients. There are, however, different factors and approaches discussed which may also play a role in the axonal targeting to the OB. The role of these factors in the signal transduction cascade can be studied using knockout experiments. The knock-out of adenylyl cyclase 3 (AC3) for example resulted in disturbed axon wiring [28]. The formation of ectopic glomeruli was seen when the tested OSNs were in an AC3-deficient background. Further, the formation of glomeruli was impaired even when the axons were shown to project to the correct position [28]. Other approaches studied the influence of the OR sequence itself on axonal wiring [20]. Other important factors for axonal wiring are the cell adhesion and cell surface molecules. Along the dorsal-ventrolateral axis, the segregation is established by neurons expressing the receptor Robo2 and the axon guidance cues Slit1. While Slit1 is highly expressed in the ventral bulb, the receptor Robo2 shows the highest expression pattern in dorsomedial OSNs [29, 30]. Additionally, the chemorepellent Semaphorin3F, released by dorsal axon terminals of OSNs, and its receptor Neuropilin2, being expressed in high levels by the ventral OSNs in the OE, influence together with Slit1 and Robo2 the positioning of OSN projections along the dorsal-ventral axis [23]. For the medial-lateral axis, the picture is not as clear as for the dorsal-ventral axis. It was shown that insulin-like growth factor (IGF) plays a crucial role in the innervation of either lateral or medial regions in the OB. IGF can act as a direct chemoattractant for OSN growth cones in cell culture, but the existence of IGF1 and 2 is not sufficient to solely explain the wiring of axons to either the lateral or medial OB [31]. Along the third axis, the anterior-posterior axis, the OR influences itself. There is one plausible model, which explains the restriction of OSN with a specific receptor to a specific glomerulus along the anterior-posterior axis. The intrinsic activity of OSNs seems to be essential for the guidance along this axis. One important factor is cAMP which can modulate the growth cone’s response to axon guidance cues. Interestingly, the transcription level of specific genes such as Neuropilin1 is correlated with the cAMP-mediated level. High levels of the Neuropilin1 receptor lead to an axon projection into the posterior OB, while low levels result in a more anterior projection [32]. Additionally, the cell adhesion molecules Kirrel2 and 3 as well as the repulsive Ephrin A5 and its receptor act in a complementary pattern in OSNs [22]. Hence, the different pattern of expression of each of those genes results in a specific pattern that determines the identity of the OR and the convergence of the same OR type to one glomerulus. The model suggests that the sensitivity of OSNs to cues positions the OSN axon to a glomerulus. These three mechanisms for axon guidance show how complex the organisation of the olfactory system is. Especially the mechanism of convergence of randomly spread OSN expressing the same type of OR reaching to the same glomeruli provides more detailed knowledge of the signalling-network.

### From the olfactory bulb to higher CNS regions

The OB consists of five layers, which are important for further signal transduction after the first excitatory afferent projection from the OSN axon to the primary dendritic terminals of mitral/tufted cells within the glomerular layer [33, 34]. Mitral and tufted cells both act as efferent neurons of the OB. This afferent excitatory synapse between OSN and mitral/tufted cells transduces its signal via the neurotransmitter glutamate towards different regions of the olfactory cortex (OC). Mitral cells (MCs) and tufted cells (TCs) transmit temporally distinct information to different OC targets. While TCs were described to project densely to focal targets only in anterior areas of the OC, individual MCs dispersedly project to all OC areas [35–37]. The activation of mitral/tufted cells results in a feedback inhibition involving lateral inhibition via dendrodendritic (GABAergic signal) reciprocal synapses with periglomerular cells, acting as interneurons. These mechanisms are important for the tuning of the incoming signal and lead to a sharpened specificity between high and less-activated mitral/tufted cells [36, 38]. Besides the periglomerular cells, the glomerular layer also receives signals from higher parts of the CNS via centrifugal afferent fibres, using a wide range of neurotransmitters. The external plexiform layer contains the somata of tufted cells, but also primary and secondary dendrites of mitral/tufted cells, as well as the apical part of granule cells. The mitral cell layer is a thin layer, mainly consisting of the somata of mitral cells and axons of tufted and granule cells together with the centrifugal fibres. The granule cell layer is mainly consisting of the interneuron’s granule cells, being important for the inhibitory circuit to mitral cells. They also receive input from the anterior olfactory nucleus, olfactory cortex, cells of the diagonal band, locus ceruleus and raphe nucleus, via afferent centrifugal fibres [33, 39].

Interestingly, unlike other sensory systems, the olfactory pathway does not pass the thalamus before reaching the cortical regions. From the OB, the axons of mitral and tufted cells form the lateral olfactory tract transmitting the signals directly to the olfactory cortex. The olfactory cortex is a structurally distinct cortical region on the ventral surface of the forebrain, which is innervated and composed of several subregions from anterior, starting with the anterior olfactory nucleus (AON), the olfactory tubercle, the piriform cortex (PC), several amygdaloid nuclei (A) and the entorhinal cortex (EC), posterior [30, 40]. The olfactory cortex represents not only a complex system of interconnections between cortex regions and the OB but also has intercortical connections to higher brain regions such as the thalamus, hypothalamus, neocortex and hippocampus. The axonal wiring during development is even more complicated than the axonal wiring within the higher CNS and towards the OB. Since the intercortical communication between the areas within the cortex are more complex than the communication within the OB, the process of axon wiring to the areas of the brain is still a field of research.

## Olfactory epithelium uptake pathways and mechanisms

It has been known since the nineteenth century that transport mechanisms and flows between the different spaces within the nose exist [41]. But these transport mechanisms remained unexplored until increasing numbers of neurological diseases pushed the field of nose-to-brain (ntb) drug delivery forward. Only a few decades ago, in 1991, William Frey II proposed a patent for a nasal drug delivery method to treat neurological disorders in the brain [1]. Since then, many exciting basic preclinical and applied clinical studies were performed showing the great potential of nose-to-brain delivery methods to treat brain diseases such as Alzheimer’s disease [42–45]. Nevertheless, the exact mechanisms through which molecules get transported from the nasal mucosa towards the CNS are still unclear but will benefit from further drug delivery approaches using IN administration.

**Fig. 3:**
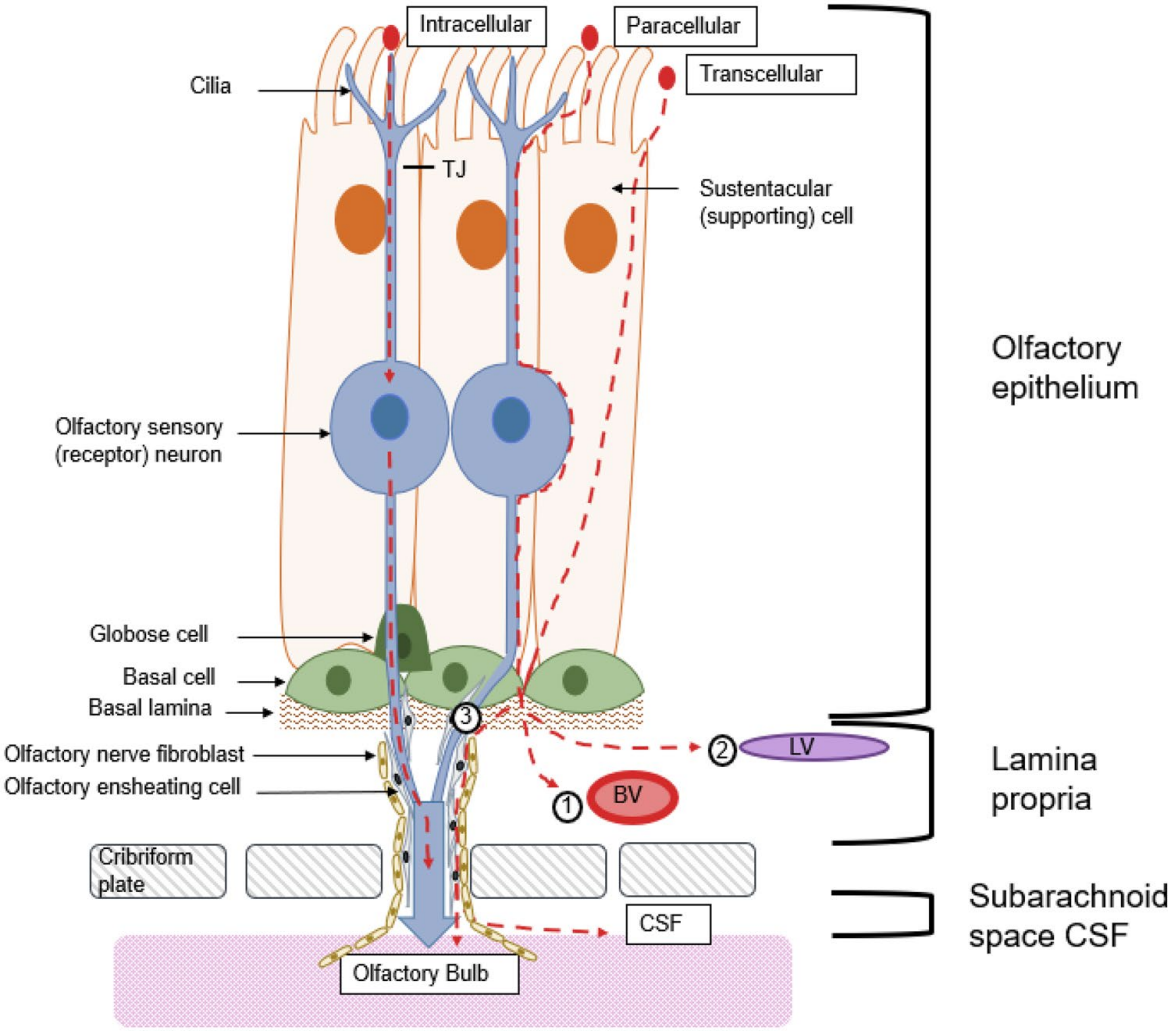
There are three different pathways through which a substance can pass the olfactory epithelium (OE). The substance can bind to a receptor and be internalised by for example receptor-mediated endocytosis. Then, it travels through the olfactory sensory neuron (OSN) towards the olfactory bulb (OB). Another possibility is that the substance uses leaky passages within the OE and travels paracellular into the lamina propria. Third, the substance can also be transported transcellular through the sustentacular cells (SUS) to the lamina propria. From there, the substance can (1) be absorbed by local blood vessels (BVs) reaching the circulation or (2) be absorbed by lymphatic vessels and be drained into the deep cervical lymph nodes of the neck. (3) The substance can use perineural spaces between the olfactory ensheating cells and olfactory nerve fibroblasts to travel associated to the olfactory nerves to the OB. After passing the cribriform plate, the substance can theoretically also reach the cerebrospinal fluid and distribute through the different brain regions

The IN administration approaches are mainly focused on two morphological structures, the olfactory and the trigeminal nerve. The pathways used by IN administered substances include intracellular, paracellular and transcellular mechanisms (Fig. 3) for the transport along the olfactory and trigeminal nerves towards the CNS. The intracellular olfactory nerve pathway uses pinocytosis and endocytosis to take up the substance into the OSN [15]. Subsequently, the substance is transported along the OSN axon to the OB. Once the molecules are delivered to the origins of the nerves in the cerebrum and pons, they can disperse throughout the brain. Another pathway is the olfactory epithelium pathway, where the substance is absorbed into the lamina propria and further enters the CNS by using the gaps surrounding the olfactory nerve tract [46, 47]. From the lamina propria, the substance can be absorbed by local blood vessels or lymphatic vessels, but most of the substances are translocated through the perineural space, between olfactory ensheathing cells and olfactory nerve fibroblasts, by bulk flow. This space leads to the subarachnoid space of the brain from where the substance can further distribute [15].

### Intracellular pathways

The intracellular pathways are used by a wide range of different molecules. Apart from the widely examined peptide insulin (5.8 kDa) [48], also gold particle (50 nm) [49, 50] and aluminium salts [51] are known to endocytose. The classic model molecule for studying intracellular pathways of the olfactory system is wheat germ agglutinin (WGA) conjugated with horseradish peroxidase (HRP) with a size of 80 kDa. In the late twentieth century, several studies showed that HRP uses pinocytosis to enter OSN [52–55]. Later, in 1995, experiments were published which indicated that in contrast to HRP alone, WGA-HRP shows an increased uptake through receptor-mediated endocytosis [56]. Even though pinocytosis seems to be more likely than receptor-mediated endocytosis, further approaches, besides WGA-HRP, indicate specific receptor expression in the olfactory mucosa, as well as in the OB. One early discovered example is the receptor-mediated uptake of the brain-derived neurotrophic factor (BDNF) [57]. Recently, many studies strongly support the view that metabolic receptors are expressed in the olfactory system and that hormones such as insulin, leptin and orexin can bind to those receptors on the olfactory mucosa [58–62].

However, viruses can use receptor-mediated endocytosis to enter the olfactory epithelium, too. Many influenza virus-subtypes, the herpesvirus and poliovirus, use different receptors on the cilia surface to enter an olfactory sensory neuron. By 1912, studies discovered the ability of the poliovirus to infect the CNS via entering the ORN [63]. In the 1930s, this hypothesis was supported by studies which finally resulted in the chemical cauterisation of the OE of Canadian school children with zinc sulphate to prevent the disease during a poliomyelitis epidemic [64–66]. Even though the poliovirus receptor was identified in the 1990s, the amount of studies focussing on the expression of the receptor on the OE surface is still rare [67–69]. Influenza subtypes can however also use structures on the cell surface to enter the olfactory epithelium. They bind to glycans with terminal sialic acid linked to galactose (SAα2,6Ga in nasal epithelium) to enter OSN [70–72]. Further, there is strong support that the herpesviruses can use heparan sulphate and nectin-1 on the cell surface to enter the olfactory neuroepithelium [73–75]. The latest example for a virus using the nose as an entry to the human body is the SARS-COV-2 virus. Recent studies showed that the nasal and olfactory epithelium expresses the obligatory receptor, angiotensin-converting enzyme II (ACE2) and the priming protease TMPRSS2. Interestingly, loss of sense of smell is an early marker for a SARS-COV-2 infection. It is proposed that the damage of sustentacular cells which express, as non-neuronal cells, ACE2 leads to the olfactory deficits in COVID-19 patients [76–79].

After internalisation of a molecule, drugs, viruses or other substances, the cargo containing vesicles (endosomes) traffic down the soma. Some pass the Golgi apparatus, while others are directly transported down the axons towards the OB. Intracellular axonal transport was visualised by using labelled tracers, showing that anterograde and retrograde transport mechanisms are present [55, 80]. The vesicles have to cover an intracellular distance within the olfactory nerve of about ~4 mm and about ~20 mm [81] within the trigeminal nerve in rats to reach the CNS. Neuronal transport is discussed to be a rather slow process. Crowe et al. state that the olfactory nerve axonal transport alone should only take 45 min for an intranasally administered drug to reach the brain [15]; however, other processes such as endocytosis and exocytosis are not included in these calculations and thus prolong the time in practice. This is based on velocity values measured in an ex vivo study using WGA-HRP and olfactory C-fibres. Two values were estimated, a slow rate with 36 mm per day, and a fast transport mechanism with 130 mm per day [80]. The fast velocity rate has been supported by further studies [53, 81]. As previously mentioned, some molecules and viruses can be endocytosed and translocated along the trigeminal nerve, which innervates the nasal cavity with two of its three main nerve divisions (V1 ophthalmic nerve; V2, the maxillary nerve) [81, 82]. Besides WGA-HRP, IN administration studies also showed that IGF-1 and the herpes simplex virus can use the trigeminal nerve pathway to reach the CNS [83–86].

### Extracellular pathways

The extracellular pathways start with either the transcellular uptake into supporting cells or paracellular diffusion through leaky parts of the nasal epithelium. Even though the epithelial cells are connected via tight junction (TJ) and express proteins such as zona occludens (ZO)-1, 2, 3, occludins and claudin-1, 3, 4, 5, 19, it is known that intercellular clefts exist [87, 88]. The nasal epithelium undergoes a constant turnover with an average lifespan of OSN of 30–60 days until they are mined by apoptosis [89–91]. As long as the OSN is not fully replaced and the TJ are not functional, there are channels remaining through which also proteins and peptides such as insulin (5.8 kDa), IGF-1 (7.5 kDa), albumin (65 kDa) and even stem cells can reach the CNS [92–96]. There are signalling mechanisms and formulation approaches to increase TJ permeability to improve the passage of drugs in the nasal epithelium. Besides various signalling regulators such as protein kinases, mitogen activated protein kinase, myosin light chain kinase and others [97], also surfactants such as bile salts and cationic polymers such as the chitin derivate chitosan are known to increase the permeability of TJs and absorption of drugs [98].

From the lamina propria, the substance travels three alongside different ways to further distribute. First, it can be absorbed by local blood vessels and enter the systemic circulation. Although the nasal epithelium, in particular the respiratory parts, is rich of vasculature, IN insulin studies support the view that the absorption in local vessels and systemic distribution after IN administration is no significant factor in the nose [44, 99]. Further, substances and molecules can be absorbed from the submucosa into the lymphatic system. The lymphatic vessels in the nose drain towards the deep cervical lymph nodes of the neck [100, 101]. The major aspect of intranasally administered molecules seems to be extracellular diffusion and convection into perineural and perivascular spaces, having a connection to cranial compartments. As mentioned previously, the intracellular axonal transport is a rather slow movement with calculated times of 0.74 (fast)–2.7 h (slow) for the distance of ~4 mm within the olfactory nerve and 3.7 (fast)–13 h (slow) for ~20 mm within the trigeminal nerve. Instead, extracellular diffusion along the olfactory and trigeminal nerve, as it takes place in the perineural space, is slightly faster. Lochhead and Throne calculated times of 0.73–2.3 h for the olfactory nerve, using a simplified version for only one dimension (distance) of Fick’s second law [15, 81]. These velocities, however, do not match experimental data, as they predict slower transport. IN studies using [^125^I]-labelled IGF-1 suggest that the rapid distribution towards the CNS (~30 min) is rather due to extracellular convection than diffusion, or intracellular transport [92]. One mechanism which could explain the rapid rates is bulk flow in the perivascular spaces in the nose. Many arteries along the olfactory nerve supply the axons with nutrients. The systolic waves result in high-pressure waves within the perivascular space. The so-called perivascular pump is suggested to be responsible for the rapid distribution of fluids and molecules towards the brain [102–104]. Calculation of the time needed for intranasally applied [^125^I]-labelled IGF-1 with a velocity of ~200 μm/min resulted in a predicted time of 0.33 h for IGF-1 to reach the brainstem via perivascular bulk flow [81]. These are predictions and do not fully represent the situation in vivo, but the predicted values support the hypothesis that extracellular transport is faster and probably the most prominent pathway for drugs to reach the CNS.

## Current status of approved drugs for nasal application

**Fig. 4:**
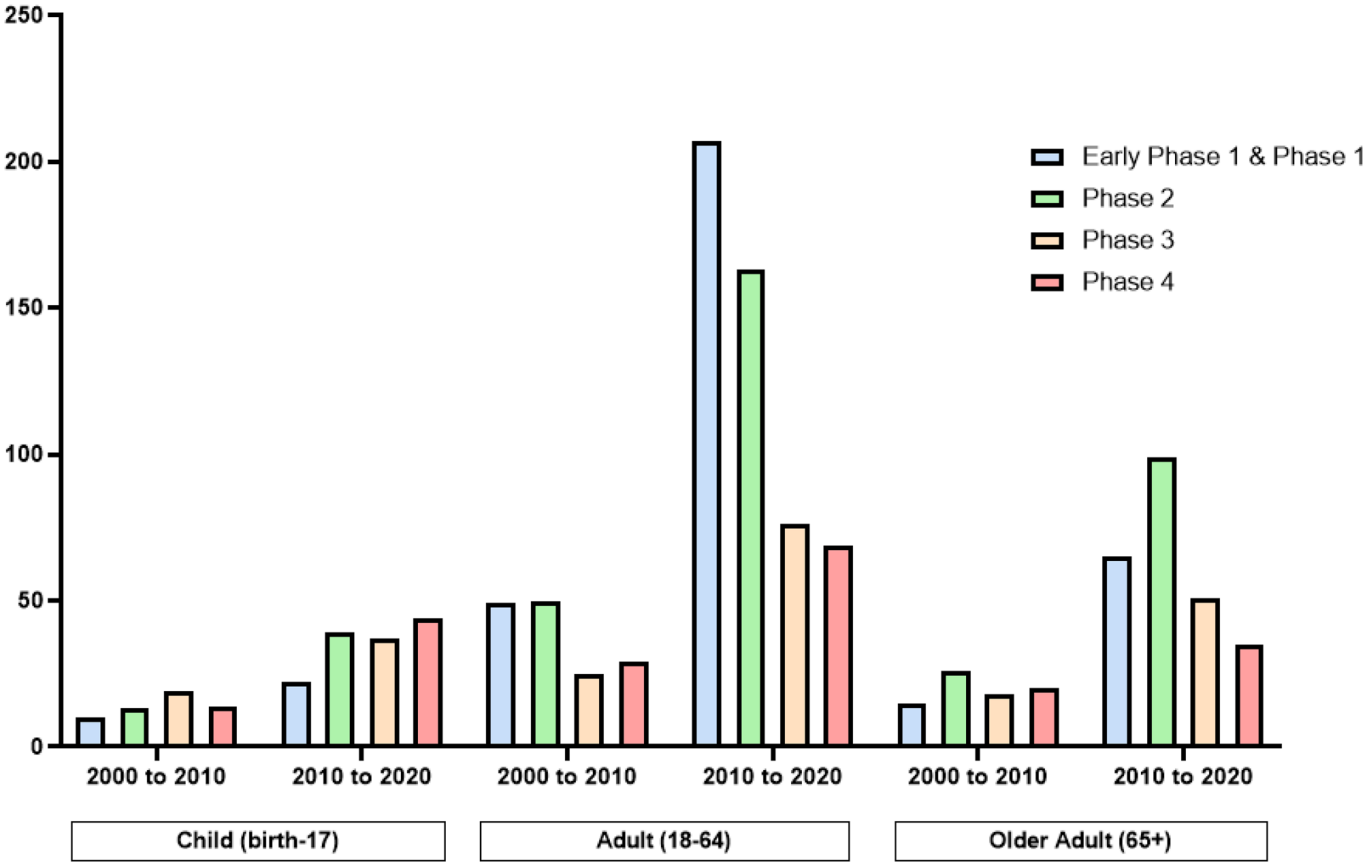
Number of clinical trial papers published at ClinicalTrial.gov using intranasal administration. Filters used contained the recruitment status (recruiting, active (not recruiting), completed); eligibility criteria (differentiated after age groups (child, adult, older adult), all sex); study results (all); study phase (early phase 1, phase 1, phase 2, phase 3, phase 4)

The IN-delivery route has gained more interest in the delivery of various drugs and treatments. In the past decade, the amount of interventional IN studies, including patients of all age groups, all sex and overall clinical phases increased around threefold compared with the time period from 2000 to 2010 (ClinicalTrial.gov) (Fig. 4). This increase in number of clinical studies is further higher than for other administration routes. There are around 18 times more completed intravenous interventional studies listed at ClinicalTrial.gov from 2000 to 2020, compared with completed intranasal interventional studies; however, the number of published interventional intravenous studies, using the same filters, only increased twofold in the past decade*.*

Even though IN delivery is usually associated with locally acting drugs, also systemic effects and treatment of CNS disorders are increasingly focussed upon by preclinical research and the pharmaceutical industry [14, 47, 105, 106]. The nasal route offers many advantages over common routes such as the oral route, as it is non-invasive and easily accessible for the administration of drugs. Further, IN administered systemically acting drugs are rapidly absorbed and exhibit a fast onset of action. The rapid absorption is due to the previously mentioned rich vasculature in the submucosa. The avoidance of the metabolic first pass effect can lead to a higher bioavailability [14, 106, 107]. It is also known for drugs intended to reach the brain that the side effects are in many cases reduced, because nasal drug delivery leads to a lower systemic exposure and a more efficient targeting [108]. One of the most prominent arguments supporting the increasing interest in IN drug delivery is the possibility to bypass the BBB. The BBB is a limiting factor for treating neurological CNS disorders [47, 109]. There are many additional limitations and aspects which have to be considered for the nasal application of drugs and biopharmaceuticals. While small, lipophilic drugs (MW < 1 kDa) are well absorbed; the nasal mucosa exhibits poor permeability for big hydrophilic drugs (MW > 1 kDa) such as peptides and proteins. Additionally, processes such as the mucociliary clearance, enzymatic degradation and a low drug retention time limit the efficiency of drug delivery via IN delivery systems. Many technologies and approaches which aim to decrease these limiting effects are under investigation [106, 107]. Nasal device technologies are under optimisation for precise targeting of the location of action and therapeutic effect. In the following sections, the terms local delivery, systemic delivery and CNS delivery are used as terms for the distribution route of a substance or drug after IN application, but not as terms for the indication.

### Intranasal application used for local delivery

Nasal delivery of locally acting drugs is commonly administered as liquid formulations via nasal sprays/aerosols pumps [110]. This delivery system is convenient as it has an additional humidifying effect and offers the possibility to reach a wide drug distribution within the nasal cavity, as it is required for local and systemic applications [111]. However, main limitations of water-based formulations and long-term usage is their microbial stability and the presence of preservatives, which can lead to irritation and allergic effects [110, 112]. Approaches are constantly developing delivery technologies, which are increasing the retention time on the mucosal surface and are reducing the variability within the patient’s individual administration procedure, for example the patients head position. Besides others nasal pressurised metered-dose inhalers, containing filters are used to apply budesonide to the anterior parts of the nasal cavity where the non-ciliated regions are the major site of deposition [112].

Popular drugs for nasal delivery are antihistamines and corticosteroids being the first-line medications to treat seasonal rhinitis and nasal congestion based on allergic reactions or infections [14]. Topically acting drugs offer advantages in these indications since they have a rapid onset. Further, antihistamines and corticosteroids have as topically acting drugs a low systemic bioavailability and hence lead to less adverse side effects and no CNS effects. Compared with oral application, the IN administration of these drugs requires lower doses [113, 114]. The role of histamines as mediators for allergic rhinitis has been known since the early twentieth century. Levocabastine is an H1-receptor–selective drug with high potency. It belongs to the second generation of histamine H1-receptor antagonists. Those second-generation drugs show an improved benefit/risk profile without CNS sedative effect when administered locally instead of orally [115, 116]. Another group of topically administered drugs for the treatment of seasonal rhinitis is the steroidal anti-inflammatory agents, namely corticosteroids. Corticosteroids bind to a single glucocorticoid receptor, localised in the target cell. Through transactivation or transrepression, anti-inflammatory effects are generated. Those effects include influencing the release of cytokines and mediators as well as a reduced inflammatory cell recruitment within the nose [117]. Many approved corticosteroids are on the market, such as budesonide, flunisolide, fluticasone, mometasone and triamcinolone. Even though corticosteroids are regarded to be more potent and efficient than antihistamines, no significant differences have been found for these two groups regarding the benefit/risk profile [113].

### Intranasal application used for systemic delivery

Apart from IN administration for local delivery of drugs, which aims to achieve local effects, IN delivery systems also aim to result in systemic exposure to achieve a wide range of therapeutic effects. IN application for systemic exposure and thus systemic delivery is under investigation in a wide range of indications such as migraine, headache, infection prevention, pain management, hormone replacement therapy, smoking cessation and emergency therapy, like for epileptic seizures [9, 98]. These approaches are due to advantages of IN application for systemic delivery. The nasal mucosa offers a relatively large surface area for drug absorption; however, the individual absorption and bioavailability values really depend on the compound, the drug formulation, the species tested in and the delivery device itself. Costatino et al. summarises that for low-molecular weight drugs, the bioavailability after IN administration is relatively high and the variability low, while for high-molecular drugs, the bioavailability is low and the variability high, compared with injections [114, 118]. For systemic delivery after IN administration, the high vascularity within the nasal cavity is a particular advantage. Further advantages are a rapid drug onset, no first-pass metabolism and non-invasiveness to maximise patient comfort and compliance [107, 119]. Historically, as it is for local delivery also for systemic delivery, most approved intranasal drug products are delivered through nasal sprays/aerosols devices [14, 112, 120]. For systemic delivery indications however, one aim of nasal device investigations is to prolong the retention time of the drug on the nasal mucosa. Therefore, to mention a few approaches, nasal gels, microemulsions/suspensions and microspheres are under development [112].

Feasibility of systemic delivery via IN application is proven for drugs used for cardiovascular indications, as some popular examples such as propranolol, nifedipine and nitroglycerin showed in the clinic [114]. Intranasally administered propranolol was shown to improve the exercise tolerance of patients suffering from angina pectoris [121]. Further, clinical studies were performed which showed that IN administration and systemic delivery of cardiovascular drugs are suitable alternatives because they result in a more rapid onset of action compared with the commonly parenteral administered formulations [121, 122].

Apart from achieving systemic effects, delivery via the nose is also used to target the CNS after reaching systemic exposure. One example for CNS targeting via systemic delivery after IN application is the administration of analgesics.

IN analgesics are used for indications such as headache and migraine treatments, as well as cancer pain. The main advantage of IN analgesics is bypassing the metabolic first-pass effect which leads to an increased bioavailability and a rapid onset. In particular, morphine, used for breakthrough pain in chronic cancer patients, underlies an extensive first-pass effect, when given orally; thus, IN delivery offers rapid and effective relief [123, 124]. Morphine is a polar, hydrophilic, low molecular weight drug. Nasal administration of morphine without any absorption enhancers results in 10% bioavailability, while the latter can be increased up to 80% for morphine in humans using chitosan-based formulations [123]. The same is true for migraine and headache treatments with IN drugs such as butorphanol, fentanyl, sumatriptan and zolmitriptan [105, 114]. The approved intranasal drug products of these small molecules do not contain nasal absorption enhancers because the drug molecules are characterised by high permeability and low molecular weight (< 1 kDa) which allows them to reach therapeutic levels in the systemic circulation. For example, the marketed drug formulation of butorphanol tartrate is delivered as an aqueous solution nasal spray containing sodium chloride, citric acid and benzethonium chloride (pH 5.0) [105, 125]. Other examples for IN application of systemic acting small molecules are analgesics with anti-inflammatory effects such as indomethacin and ketorolac [14].

Feasibility of IN drug delivery with systemic dispersion for sedative agents and in emergency situations such as epileptic seizures (benzodiazepines) or for opioid overdoses (naloxone) is under investigation. Nyxoid® is for example the nasal formulation of naloxone approved in Europe. The formulation contains naloxone hydrochloride dihydrate, trisodium citrate dihydrate, sodium chloride, hydrochloric acid, sodium hydroxide and purified water [126]. The benzodiazepine midazolam is used intranasally for both indications. In clinical trials, its use has not only been described as a sedative agent but also as an emergency pre-hospital treatment for antiepileptic medication [114, 127, 128]. For the application via the IN route, both indications benefit from the rapid onset and improved bioavailability, compared with the oral route and easy handling, which is in particular relevant for caregivers in pre-clinical situations [129, 130].

The replacement of hormones with an IN delivered drug is an important indication for IN application with systemic delivery. One example is the aqueous formulated 17-β-estradiol (Aerodiol®), which is used as an oestrogen therapy to reduce menopause symptoms in women. Studies have shown that IN compared with oral and transdermal administration resulted in similar area under the plasma concentration time curve (AUC) up to 24 h, efficacy and frequency of adverse effects [131–134]. Furthermore, biomacromolecules such as peptides and proteins are marketed for hormone replacement therapies. For instance, nasal sprays containing salmon calcitonin (Miacalcin®, Novartis; Fortical®, Unigene) are available on the market to treat osteoporosis; desmopressin (Desmospray®, Ferring) is used IN as a antidiuretic hormone to treat diabetes insipidus, enuresis, haemophilia A and the von Willenbrand’s disease (type I), and gonadotropin-releasing hormone analogs such as busrelin (Suprecur®, Sanofi-Aventis) and nafarelin (Synarel®, Pharmacia) are used to reduce the testosterone and oestrogen levels in blood of patients suffering of prostate cancer and endometriosis [105, 135–140]. None of these products contain any absorption enhancers, even though systemic bioavailability of peptides and proteins after IN application is usually below 1%. However, these mentioned peptide-based products are effective as they are potent and therapeutic even at low systemic levels [125].

IN delivery is in general increasingly being used as a delivery route for small but also for biomacromolecules treating systemic indications. Nevertheless, limitations such as the fast-mucosal clearance and low absorption capacity of the nasal mucosa for non-aqueous formulations have to be resolved by improving the composition of excipients and absorption enhancers within the formulation.

### Intranasal application used for CNS delivery

Oral drug delivery is the most common way to administer a drug to humans. However, for treatment of neurological disorders or diseases, oral delivery methods often fail to deliver drugs efficiently to the central nervous system (CNS). Barriers exist in the brain, most importantly the BBB, which protect the CNS from pathogens, neurotoxic molecules and other potentially harmful substances. The BBB is an essential interface between CNS and periphery and is composed of endothelial cells, which are tightly connected to each other by TJs and adherents junctions (AJs) [119, 141–143]. Another factor reducing the amount of substance reaching the CNS via the systemic route is the presence of multidrug efflux protein transporters. The active P-glycoprotein efflux pumps are found at the BBB luminal side and reduce the amount of drug exposure in the CNS, by expelling them back into the bloodstream [142, 144]. Studies showed that the olfactory system contains such efflux transporter [145], too. However, only small drug molecules (< 500 Da) of high lipophilicity are able to cross the BBB which accounts only for < 1% of macromolecules and only 2% of small molecules [108, 141, 146]. In the context of treating neurological disorders such as AD, the IN administration of drugs gains more and more interest, because it bypasses the BBB and the systemic first-pass effect and is therefore a promising approach as an efficient drug delivery route [47, 119].

As described previously, a direct path from the OE to the CNS exists. This path is in association with intracellular and extracellular pathways, involving transcellular, paracellular and extracellular transport mechanisms through which drugs can be transported from the nasal mucosa to the CNS alongside the olfactory and trigeminal nerve fibres. Studies showed that not only small molecules but also peptides and proteins use these pathways to directly reach the OB, being a forebrain structure. From the OB, they can also either disperse extracellularly or they are further transported intracellularly towards higher brain regions [81, 146]. Regardless of the administration route, achieving constant and targeted delivery in the brain parenchyma is still challenging because substances have to travel long distances within the brain (~ mm) to reach their target [147, 148]. Transport within the extracellular space plays a critical role for the diffusion of drugs, as it makes up ~ 20% of the brain volume [148, 149]. No matter if a drug or substance reaches the brain after intranasal delivery, crossing the BBB or direct infusion into the brain, the distribution within the microenvironment involves extracellular diffusion [148]. Brain distribution can be facilitated by nanotechnology [150]. Studies have shown that nanotherapeutics and nanomaterials improve the biodistribution of drugs in the brain for more efficient treatment of glioblastoma, not only via convection-enhanced delivery [151] but also via intranasal delivery [152].

Chemotherapeutic agents are in general considered for INDD systems [152, 153]. The main advantage of the IN route is the chance of reduced side effects in other organ systems [146, 154]. Different approaches showed that chemotherapeutic agents for treating brain tumours such as perillyl alcohol, methotrexate and telomerase inhibitors can be delivered via the nasal route and are effective alternative strategies [155–160].

Further, several peptides and proteins are under investigation for ntb delivery. Prominent examples include oxytocin known for its positive effects on social behaviour and autism [161–163] as well as orexin-A improving the CNS hypocretin signalling and olfaction and thus offering a possible treatment for narcolepsy [164–166]. Another potential agent for nasal drug delivery is leptin for treating obesity and sleep disorders. While systemic and peripheral administration of leptin failed to lead to a positive effect, preclinical IN leptin studies showed a reduced appetite and resulting weight loss [167–169].

An example for illustrating the feasibility of IN application of drugs targeting the CNS via olfactory-associated pathways is insulin. Insulin is an important regulator for the energy metabolism in the CNS. Insulin-sensitive glucose transporters transport insulin across the BBB, while insulin receptors are widely expressed in different regions of the brain, with highest concentration in the OB, cerebral cortex, hypothalamus, hippocampus and cerebellum [170, 171]. The perturbation of its function in the CNS and the shift towards lower insulin concentration in the brain (or CSF) to the periphery are shown to contribute to the formation of cognitive deficits and Alzheimer’s disease. In addition, peripheral abnormalities such as hyperglycaemia and the diabetic state are improving the risk to develop AD [42, 172–174]. This suggests that patients suffering from diabetes are more likely to develop AD. In turn, many studies, preclinical and clinical, show the ability of IN insulin to improve cognition and memory in age-related cognitive deficits [43, 175–178]. Still, no approved formulation for IN administration of insulin is on the market.

RNA therapeutics have also to be mentioned and are in the focus for IN delivery to treat neurodegenerative diseases. There is an increasing number of publications in the last decade investigating the potential of siRNA and anti-sense-oligonucleotides for such approaches, as it was shown that, for example, siRNAs travel along the olfactory nerve after IN delivery [48, 179–182].

Another exciting new application for IN application is the delivery of stem cells along the olfactory tract towards the CNS. In 2009, a group of scientists first suggested that fluorescently labelled rat mesenchymal stem cells (MSCs) and human glioma cells intranasally applied to naive mice and rats use the olfactory-associated neuronal pathways to reach different regions in the CNS [183]. Later, the potential of IN applied neuronal stem cells/progenitor cells carrying bioactive gene products to target intracerebral glioma was elucidated [184]. Currently, the number of pre-clinical studies using neuronal stem cells, progenitor cells or mesenchymal stem cells to investigate their potential to treat brain tumours or neurodegenerative diseases is rising. These approaches seem to offer a safe and efficient alternative to the surgical injection or intravascular administration [185–188].

The approval of drug formulations for CNS delivery through olfactory tract–associated pathways is, however, depending on appropriate nasal devices, which are increasing the drug distribution and absorption through the olfactory epithelium. The distribution pattern of nasal formulations strongly depends on the particle size, which in turn is affected by the administration device and the physiochemical properties of the formulation. The viscosity of the formulation influences the droplet size of a nasal spray and thus its deposition site [189]. Higher viscosity of nasal formulations enhances the absorption through the nasal mucosa because of prolonged retention time. Conversely, it reduces systemic delivery because of slower diffusion [190]. In this context, great efforts are made to develop new delivery strategies such as nanotechnology-based approaches and devices such as bi-directional nasal insufflators which facilitate the distribution to the posterior part of the nose and minimise lung deposition [112, 191]. A recently published review from Rabiee et al. [45] summarises and discusses existing approaches using natural and polymeric nanoparticles for ntb delivery. The authors conclude that polymeric nanoparticles are promising carriers for ntb delivery of drugs against Alzheimer’s disease. This conclusion, however, may be also applicable for other CNS indications.

## Toxicological challenges of intranasal application

Safety is a key issue when designing an effective and safe drug formulation, for IN administration. During the development process, safety consideration not only of the drug itself but also of the active ingredients and excipients within the formulation must be considered. Absorption enhancers are necessary for large molecules such as peptides and proteins. They increase the bioavailability of the drug following IN administration by improving the permeability of the nasal mucosa. Other excipients act as mucoadhesives and prolong the contact time with the nasal mucosa. Because of their own safety profile and the increased local exposure time of the drug, the excipients can significantly decrease the safety of the final drug product [192–194]. Also, the toxicological considerations must be discussed regarding local, systemic, CNS and pulmonary effects of the drug formulation.

### Local side effects

The local tolerability of a drug product depends on many different factors and differs between individuals. Environmental cues such as temperature and humidification, psychologic factors but also individual physiological factors such as infections, pre-existing illness or allergies influence the local interactions between drug product and nasal mucosa. For this review, only intrinsic biologic factors are considered. Those biological factors are affecting the drug absorption in the nasal mucosa and therefore influence the toxicologic profile of the final drug product. The nasal blood flow regulates important conditions in the nose such as temperature or humidification of inhaled air. There is a range of drugs that are known to influence the blood flow, such as vasomotors. Oxymetazoline, which is used as a decongestant for allergies and colds, was shown to decrease the blood flow within the nose as a vasoconstrictor [195–197]. Further, IN corticosteroids are also vasoconstrictors leading to relief in patients with seasonal rhinitis. Rare side effects such as nose bleeding and the very rare occurrence of nasal septal perforation were observed [113, 115, 198, 199]. In contrast, other drugs increase the blood flow in the nose, for example histamine, albuterol, isoproterenol and fenoterol [113, 118].

Another biologic factor which should be considered regarding toxicologic and safety issues is the enzymatic activity in the nasal mucosa. As the nasal mucosa is a direct contact zone towards environmental keys, it also represents a barrier towards harmful substances and xenobiotics. Hence, there are also defensive enzymes present that metabolise substances and drugs. To date, it is known that the nasal mucosa has a wide spectrum of xenobiotic-metabolizing enzymes, comprising enzymes belonging to the P450-dependent metabolism pathway (e.g. P450 monoxygenase), Phase I enzymes (flavin monooxygenases, aldehyde dehydrogenases, epoxide hydrolases, carboxylesterases, etc.) and Phase II enzymes (glucuronyl and sulphate transferases, glutathione transferase) [118, 200, 201]. It can be assumed that these enzymes are also metabolizing intranasally administered small-molecule drugs such as opioids, histamines, corticosteroids and more [118, 202].

Not only enzymes protect the nose and upper airways from potentially harmful substances and xenobiotics, but also the nasal mucociliary system represents a major part of defence mechanisms within the nose. The mucus layer covers the nasal epithelium and transports particles through ciliary beating towards the nasopharynx. The ciliary beating frequency (CBF) is under cellular control, regulated by temperature, intracellular Ca^2+^, cAMP and extracellular ATP level. Other physiological functions of the nasal mucosa include its water-holding capacity and its responsibility for the efficient heat transfer within the airway. Further, it exhibits surface electrical activity [193]. Thus, the impairment of these systems can lead to longer contact times of formulations, physiologic impairment and damage of the mucosa and the nasal epithelium.

The human nasal mucosa has an average physiologic pH of 6.3 and is therefore slightly acidic. The maintenance of the pH in the mucus ensures the function of the ciliary clearance [203]. Therefore, the pH of nasal formulations should be within a pH range of 4.5 to 6.5 to avoid nasal irritation [118]. Not only the pH but also the osmolarity has an influence on the ciliary beat and can therefore contribute to local toxicological considerations [118, 190]. Many substances are however influencing the mucociliary clearance (MCC) through either stimulation or inhibition. Instead of stimulation effects, the inhibitory effects are the main cause of adverse side effects such as nasal dryness, irritation, sneezing, nasal itching but also rhinitis medicamentosa and congestion. It is worth to mention that MCC and CBF effects are usually evaluated in vitro. Those in vitro tests do not allow predictions about ultimate effects in vivo, as in vitro tests show effects on MCC and CBF, whereas in vivo the same compounds often do not lead to detectable side effects [204]. In general, it was shown for many compounds that the inhibitory effect on the mucosal clearance and CBF is dose and time dependent. For example, the α-adrenergic receptor agonists oxymetazoline and xylometazoline showed inhibitory effects for human nasal mucosa in vitro in a dose-dependent manner [205, 206]. Many corticosteroids and anti-histamines are also influencing the MCC and CBF in in vitro studies but at the same time show no adverse effects in vivo [194, 207–209]. The mucociliary effect of drugs is however only one aspect. Excipients are used not only to improve the drug transport and bioavailability through the nasal mucosa and epithelia but also to protect the drug product from microbial contamination and degradation. Those enhancers and preservatives have to be considered and evaluated in toxicological examinations. A prominent example for the toxicologic relevance of preservatives is benzalkonium chloride (BKC), which is used for cosmetics and in several nasal formulations. BKC showed in different animal models such as chicken embryo tracheas, rat and guinea pig tracheal tissue the inhibitory effect on CBF. This effect is dose and time dependent with ciliostasis and ciliotoxicity as the ultimate response [210, 211]. In vivo histological examinations in rats showed that BKC can also provoke nasal lesions. Concentrations of 0.05 and 0.10 w/v % BKC administered into the nasal cavity of rats led to histopathological findings such as epithelial desquamation, degeneration, oedema or neutrophilic cellular infiltration in the anterior parts of the nasal mucosa [212]. Further studies support the toxic effect of BKC on the nasal mucosa in vivo [213]. For example, in one study, 10 µl of nasal steroid formulations was administered twice daily to rats for 21 days, either with or without BKC (310 or 220 µg/ml). In the nasal cavities of rats receiving a formulation containing BKC, a range of alterations including reduced epithelial cell high, pleomorphism of individual epithelial cells, reduced number of cilia and goblet cells associated with a loss of mucus covering the epithelial cell layer was observed [214]. Further, in in vitro studies, the toxic and CBF inhibitory effects on human nasal mucosa were observed as well [207, 215]. Nevertheless, the safety concern about BKC remains controversial, as there are studies reporting no toxic effect of BKC in vivo. However, the use of BKC in aqueous formulation in vivo has been considered as safe [194, 213, 216]. The European Medicines Agency (EMA) summarises that the average nasal use of BKC in medicine products is between 0.02 and 0.33 mg/mL and that preclinical data show a time- and concentration0dependent toxic effect on cilia in vitro and in vivo in rats. Further, they state that it is not possible to recommend any safety limit for the general population of patients [217].

Besides preservatives, also penetration enhancers are used in nasal formulations. They are improving the bioavailability and transport of compounds across the nasal epithelium and mucosa. Wanted effects of enhancers include opening of TJs, alteration of the mucus layer and inhibition of proteolytic enzymes. In turn, those functions have a disruptive character and can thus lead to adverse side effects, which can be additive [192, 194, 204, 216]. It is essential to consider that many substances and compounds act as irritants to the nasal mucosa but are non-damaging. The local effects of a formulation are always an interplay between drug and excipients. Further, the testing procedure such as dose, time and in vitro test system as well as animal species has to be evaluated carefully before concluding about safety issues.

### Systemic and CNS side effects

One of the main advantages using INDD compared with other administration routes such as oral or intravenous application is the bypassing of the metabolic first-pass effect and the reduced risk of systemic adverse effects. Intranasal 17 b-estradiol, marketed as AERODIOL, is an example for the possible superiority of IN drug delivery over oral drug delivery. Several clinical studies showed that AERODIOL leads to less systemic adverse side effects, such as mastalgia and breakthrough bleeding, compared with oral or transdermal delivery, while exhibiting at least the same efficiency [132–134, 218]. The same was observed for intranasally delivered benzodiazepines such as diazepam and midazolam, which are used to treat seizures and epilepsy in emergency situations besides other indications. Major observed systemic side effects include not only sedation, drowsiness, sleepiness or amnesia, but also respiratory depression is a potential side effect [219]. However, clinical and pre-hospital studies are supporting the view that IN benzodiazepines are as safe or safer than oral, rectal or intravenous administration [220, 221]. Many systemic side effects and effects on the CNS are the result from the ability of the substance to reach the blood circulation and traverse the BBB. In conventional epilepsy treatments, drug resistance to anti-epileptic drugs can occur when drug does not pass the BBB sufficiently, as a result from improper dosing and wrong drug choice [222]. These complications, as well as drug-associated toxicities, can be overcome by suitable drug delivery systems. Nanotechnology-based systems are a rising technology for improving ntb delivery. They facilitate a more targeted and efficient brain delivery and reduce side effects at the same time [223]. The advantage of using nanotechnology-based drug delivery systems has been shown for different CNS indications, such as epilepsy, psychosis-related disorders and glioma [152, 223–227].

The nasally administered sympathomimetic oxymetazoline is used not only as a topical treatment for rhinitis but also as an anaesthetic and for the treatment of epistaxis. Oxymetazoline is a potent peripheral alpha adrenergic 1 and 2 agonist, but when it reaches the systemic blood circulation, it can also stimulate central alpha 2 adrenoreceptors. Hence, adverse systemic effects include, amongst others, vasoconstriction and sympathetic effects such as fast, irregular or pounding heartbeat, headache, dizziness, drowsiness, high blood pressure, nervousness and trembling [197, 228, 229]. These effects can cause hypertension, tachycardia and peripheral vasoconstriction. However, the adverse side effect profile of intranasal oxymetazoline is not unique and aligned with the side effects of sympathomimetics in general, regardless of the administration route. Further, those side effects are in particular relevant in paediatric medicine and for patients with underlying medical conditions [228–232].

Intranasally administered drugs and formulations show an overall better systemic tolerability compared with other administration routes such as intravenous or oral application. This is mainly due to bypassing of the metabolic fist-pass effect. Further, systemic adverse effects observed in the clinic, such as drug resistance to anti-epileptic drugs, depend on the properties of the drug itself, wrong handling or overdosing but not on the administration route [222].

### Pulmonary effects

Drug or substance-induced respiratory and pulmonary problems are intensively described in clinical and histological observations. They range from mild effects such as coughing or breathing problems during sleep to severe effects such as pulmonary toxicity, infections, pneumonia and acidosis. Over 1300 substances and drugs are listed to affect the respiratory tract (www.pneumotox.com). In this review article, we will only focus on drugs inducing adverse respiratory effects after IN administration. As described above, benzodiazepines are applied intranasally to treat emergency seizure events in paediatric populations. One known adverse side effect is treatment-induced respiratory depression. This side effect is, however, independent of the administration route. In fact, collected data suggests that IN delivery of benzodiazepines such as midazolam and diazepam is safer with regard to respiratory depression compared with oral or intravenous administration. It is judged to be as safe as rectal administration [219, 233, 234]. Another example is the neurohormone oxytocin, which is used intravenously for labour induction, for abortions or for the control of post-partum bleeding. Even though oxytocin is described as a relatively safe medication, rare cases of treatment-induced severe adverse and life-threatening side effects such as pulmonary hypertension and pulmonary oedema were reported [235–238]. Intranasally delivered oxytocin is under investigation to treat psychiatric disorders. There are a number of IN oxytocin studies in humans; however, adverse events are not described according to a standardised scheme and are therefore inconsistently reported [161–163, 239, 240]. Since IN delivery is not a popular administration route, studies comparing effects on safety of IN vs IV administration of oxytocin are rare. Literature reviews that report safety data of IN oxytocin administration have only mentioned mild respiratory effects such as asthma attacks [241, 242].

Pulmonary effects can be an important reason for a drug formulation to get delayed market approval or even withdrawn after approval. This was a lesson learned for the field of pulmonary drug delivery. Exubera® was the first approved formulation of inhalable insulin from Pfizer Labs (New York, NY), which reached the US market in 2006. It was used to treat type 1 and 2 diabetes in non-smokers without pulmonary diseases [243]. Apart from economic reasons for withdrawing Exbuera®, it showed pulmonary toxicity issues [244]. During clinical use, symptoms such as non-progressive dry cough were observed, and pulmonary function test became necessary, as pulmonary function parameters decreased during long-term use [243, 245]. Further, there were some cases of previous smokers treated with Exbuera® who developed lung cancer. Insulin is a growth factor, and inhalation of insulin could lead to a secondary activation of pro-proliferative insulin-like growth factor (IGF-1) pathway [246]. There were, however, too few cases to determine whether these lung cancer cases were associated with the inhaled insulin formulation [243]. Since, 2014, the company Mannkind Cooperation received the US market approval for Afrezza, an inhaled insulin with improved PK/PD properties, but the pulmonary toxicity issue could not be ruled out after all [247].

It is important to consider that not only the drug itself but also other components of a drug formulation can result in adverse pulmonary and respiratory effects. In a dose- and concentration-dependent manner, benzalkonium chloride showed that its target organ is the lung. It induces lung irritation, inflammation and alveolar damage after inhalation and can lead to pulmonary oedema and pneumonia after oral or intravenous administration in rats [248]. Indeed, benzalkonium chloride used as a preservative in nasal sprays in low concentrations of 0.007–0.01% is considered to be safe regarding pulmonary effects [249].

## Regulatory considerations

Obtaining regulatory approval of a candidate to market is the end of a long drug development procedure, but regulatory aspects should be aware from the beginning. To get approval of any new drug product in the USA requires safety, efficacy and quality considerations. This information will be submitted as a new drug application (NDA) to the FDA. However, at the moment, orally inhaled and/or nasal drug products (OINDP) are most frequently discussed for repurposing of an already approved product, as it was previously described for several examples like inhaled insulin (Exbuera®). Therefore, this section focuses on special safety, efficacy and quality considerations for the respiratory delivery regarding requirements of the US Food and Drug Administration (FDA). Further, biologics are not separately mentioned as those considerations apply to biologicals as well as to small-molecule drug products.

There are three different regulatory pathways to get a new drug product approved by the FDA, 505 (b)(1), 505(b)(2) for NDAs and 505(j) abbreviated NDAs (ANDAs). Repurposed drugs using a new administration route like respiratory delivery can be qualified for the regulatory procedure 505(b)(2). This can be beneficial, since some safety and efficacy data could be used from previously approved drugs [250–252]. Even though preclinical studies and systemic safety could be justified by data of previous studies of approved drugs, there are still additional information required from new preclinical and clinical studies [253].

### Preclinical considerations

The FDA published a guidance document summarizing preclinical studies, which could be required for a reformulated drug product and alternate administration routes. In general, for all drug product reformulations and for all drug products with new routes of administration, the recommendations outlined in ICH M3 (R2) and ICH S9 must be followed. The study design requirements for preclinical studies for IN delivery should be similar to inhalation studies for new formulations. These studies include a short-term study (2–4 weeks) in two species (at least one non-rodent) and then followed by a chronic study with up to 6 months in the most appropriate species. If in the chronic inhalation study no toxicity resulting in proliferative or preneoplastic changes is observed and when adequate local airway exposure by the oral route in previous carcinogenicity studies was reached, then no additional carcinogenicity study is required for the inhalation route. For local safety, the guidance document includes histological assessments of local tissue (lung, nasal mucosa, bronchi) and potentially affected brain areas [254].

Salminen, Wiles and Stevens summarised in a review the nonclinical requirements for 505(b)(2) NDAs; however, they conclude that the nonclinical requirements are highly drug product–dependent [255]. For example, the question whether additional studies are needed for efficacy evaluation depends on the product’s use. For OINDPs for systemic use, it may be possible to show efficacy of the respiratory route via bioequivalence pharmacokinetics and relative bioavailability studies. For local use however, the efficacy cannot be derived from previous studies of different purpose [253].

### Clinical considerations and human factors

In the clinical area, the safety information from systemic exposure can be reused for already approved active pharmaceutical ingredients (APIs) and for excipients (inactive ingredients), but not if the drug is intended to be used for local exposure. Then, new safety information have to be generated; this is also true for formulations which use non-approved excipients [253]. Typically, Phase III clinical trials are required for a 505(b)(2) application as the efficacy of the nasal administration route has to be demonstrated. One of the biggest hurdles for nasal drug delivery devices is the interaction interface of the patient with the device. This can influence the performance and efficacy and can lead to ineffective or unsafe use. Hence, the FDA is working on guidelines for human factors engineering (HFE) and usability engineering (UE) [256–258].

### CMC considerations

In the area of Chemistry, Manufacturing and Controls (CMC), there is a substantial package of information required, containing a quality control (QC) program, as well as ‘product characterisation’ tests. The QC program for OINDPs includes for example recommendations to test delivered dose uniformity, aerodynamic particle size distribution and extractables of nasal sprays, metered dose inhalers (MDI) and dry powder inhalers (DPI) [259, 260]. The recommendation is that the applicant should develop a list of critical quality attributes early in the device development. However, the CQAs, which are physical, chemical, biological or microbiological properties or characteristics, should be in line with the desired product quality outlined in ICHQ8(R2) [260]. The CMC area is for OINDPs a special challenge as the device performance is not fixed, also for approved nasal delivery devices. The performance depends on the formulation and the patient’s interaction and has to therefore be critically evaluated.

## Conclusion

To date, IN drug formulations are mainly approved and used for local and systemic indications, not only for seasonal rhinitis and pain management but also for emergency situations such as epileptic seizures in paediatric population. The interest in INDD is however growing for CNS indications as in neurodegenerative diseases and other types of CNS disorders, because the BBB is limiting the bioavailability of drugs after systemic delivery. Furthermore, vaccine development will increasingly include nasal delivery, as the current COVID-19 pandemic, resulting in pulmonary disorders, highlights. Apart from the advantages of the non-invasive use and the possibility of more targeted dosing to reduce systemic side effects, there are still limitations and toxicological considerations regarding formulation aspects. There is a need for more basic research to understand the anatomy and physiology, but also pathologic mechanisms and their influence on each other. Elucidating the pathways and distribution routes that individual compounds take after IN administration is crucial to develop suitable drug delivery systems for IN approaches. There are more experimental approaches and studies needed, combining imaging methods in vivo (live and retrospective), with quantitative analysis of compound levels in the respective tissue or target organ. The influence of the formulation composition on the drug distribution routes and pathways has to be further studied. Formulation properties and nasal device technologies will be in focus for the optimisation for targeted delivery regarding how to favour for example ntb delivery or pulmonary delivery over systemic absorption. Based on published work, it is unclear which dose requirements would be necessary for a respective drug to achieve therapeutic levels in the brain. Hence, a systematic comparison of different formulation approaches for efficient delivery has to be investigated. Elucidating the role of olfactory and trigeminal nerves in the transport of drugs and their formulations from the nose to the brain or other parts of the body is therefore an important basic research question which will open new possibilities for therapeutic approaches. Indications requiring gene or cell therapy approaches, but also seasonally emerging pandemics and pulmonary diseases, will benefit from future optimised and robust INDD systems. The increasing number of drug products in clinical phases and the development of new nasal delivery devices will also push forward the regulatory considerations and guideline development required for market approval.

## References

[1] Frey W H.III. Neurologic agents for nasal administration to the brain [Internet]. 1991. Available from: https://patentscope.wipo.int/search/en/detail.jsf?docId=WO1991007947&tab=PCTBIBLIO

[2] Axel R (2005). Scents and sensibility: a molecular logic of olfactory perception (Nobel lecture). Angew Chem Int Ed.

[3] Crisler R, Johnston NA, Sivula C, Budelsky CL. Functional anatomy and physiology. Lab Rat [Internet]. Elsevier; 2020 [cited 2020 Jul 13]. p. 91–132. Available from: https://linkinghub.elsevier.com/retrieve/pii/B9780128143384000040

[4] Mygind N, Dahl R (1998). Anatomy, physiology and function of the nasal cavities in health and disease. Adv Drug Deliv Rev.

[5] Harkema JR, Carey SA, Wagner JG (2006). The nose revisited: a brief review of the comparative structure, function, and toxicologic pathology of the nasal epithelium. Toxicol Pathol.

[6] Chamanza R, Wright JA. A review of the comparative anatomy, histology, physiology and pathology of the nasal cavity of rats, mice, dogs and non-human primates. Relevance to inhalation toxicology and human health risk assessment. J Comp Pathol. 2015;153:287–314. 10.1016/j.jcpa.2015.08.009 26460093

[7] Jones N (2001). The nose and paranasal sinuses physiology and anatomy. Adv Drug Deliv Rev.

[8] Henson B, Drake TM, Edens MA. Anatomy, head and neck, nose sinuses. StatPearls [Internet]. Treasure Island (FL): StatPearls Publishing; 2020 [cited 2020 Jul 24]. Available from: http://www.ncbi.nlm.nih.gov/books/NBK513272/30020644

[9] Stamegna J-C, Girard SD, Veron A, Sicard G, Khrestchatisky M, Feron F (2014). A unique method for the isolation of nasal olfactory stem cells in living rats. Stem Cell Res.

[10] Girard SD, Devéze A, Nivet E, Gepner B, Roman FS, Féron F. Isolating nasal olfactory stem cells from rodents or humans. J Vis Exp [Internet]. 2011 [cited 2020 Nov 30]; Available from: http://www.jove.com/details.php?id=2762 10.3791/2762 PMC3217619 21876529

[11] Harkema JR (1990). Comparative pathology of the nasal mucosa in laboratory animals exposed to inhaled irritants. Environ Health Perspect.

[12] Treuting PM, Dintzis SM, Montine KS (2018). Comparative anatomy and histology: a mouse, rat, and human Atlas.

[13] Gizurarson S (1990). Animal models for intranasal drug delivery studies. A review article Acta Pharm Nord.

[14] Pires A, Fortuna A, Alves G, Falcão A (2009). Intranasal drug delivery: how, why and what for?. J Pharm Pharm Sci.

[15] Crowe TP, Greenlee MHW, Kanthasamy AG, Hsu WH (2018). Mechanism of intranasal drug delivery directly to the brain. Life Sci.

[16] Huart C, Rombaux P, Hummel T (2019). Neural plasticity in developing and adult olfactory pathways – focus on the human olfactory bulb. J Bioenerg Biomembr.

[17] Field P, Li Y, Raisman G (2003). Ensheathment of the olfactory nerves in the adult rat. J Neurocytol.

[18] Buck L, Axel R (1991). A novel multigene family may encode odorant receptors: a molecular basis for odor recognition. Cell.

[19] Mombaerts P (2004). Odorant receptor gene choice in olfactory sensory neurons: the one receptor–one neuron hypothesis revisited. Curr Opin Neurobiol.

[20] Mombaerts P (2006). Axonal wiring in the mouse olfactory system. Annu Rev Cell Dev Biol.

[21] Chess A, Simon I, Cedar H, Axel R (1994). Allelic inactivation regulates olfactory receptor gene expression. Cell.

[22] Serizawa S (2003). Negative feedback regulation ensures the one receptor-one olfactory neuron rule in mouse. Science.

[23] DeMaria S, Ngai J (2010). The cell biology of smell. J Cell Biol.

[24] Ressler KJ, Sullivan SL, Buck LB (1993). A zonal organization of odorant receptor gene expression in the olfactory epithelium. Cell.

[25] Mori K (1999). The olfactory bulb: coding and processing of odor molecule information. Science.

[26] Miyamichi K (2005). Continuous and overlapping expression domains of odorant receptor genes in the olfactory epithelium determine the dorsal/ventral positioning of glomeruli in the olfactory bulb. J Neurosci.

[27] Zapiec B, Mombaerts P (2020). The zonal organization of odorant receptor gene choice in the main olfactory epithelium of the mouse. Cell Rep.

[28] Zou D-J, Chesler AT, Le Pichon CE, Kuznetsov A, Pei X, Hwang EL (2007). Absence of adenylyl cyclase 3 perturbs peripheral olfactory projections in mice. J Neurosci.

[29] Cho JH, Lepine M, Andrews W, Parnavelas J, Cloutier J-F (2007). Requirement for Slit-1 and Robo-2 in zonal segregation of olfactory sensory neuron axons in the main olfactory bulb. J Neurosci.

[30] de Castro F. Wiring olfaction: the cellular and molecular mechanisms that guide the development of synaptic connections from the nose to the cortex. Front Neurosci [Internet]. 2009 [cited 2020 Jul 13]; Available from: http://journal.frontiersin.org/article/10.3389/neuro.22.004.2009/abstract 10.3389/neuro.22.004.2009 PMC2858608 20582279

[31] Scolnick JA, Cui K, Duggan CD, Xuan S, Yuan X, Efstratiadis A (2008). Role of IGF signaling in olfactory sensory map formation and axon guidance. Neuron.

[32] Imai T, Suzuki M, Sakano H (2006). Odorant receptor-derived cAMP signals direct axonal targeting. Science.

[33] Simpson KL. Olfaction and taste. Fundam Neurosci Basic Clin Appl [Internet]. Elsevier; 2018 [cited 2020 Jul 13]. p. 334–345.e1. Available from: https://linkinghub.elsevier.com/retrieve/pii/B9780323396325000232

[34] Susaman N, Altundağ A, Rombaux P. Olfactory function. In: Cingi C, Bayar Muluk N, editors. Nose [Internet]. Cham: Springer International Publishing; 2020 [cited 2020 Jul 13]. p. 71–4. Available from: http://link.springer.com/10.1007/978-3-030-21217-9_8

[35] Haberly LB, Price JL (1977). The axonal projection patterns of the mitral and tufted cells of the olfactory bulb in the rat. Brain Res.

[36] Shepherd GM (2007). Perspectives on olfactory processing, conscious perception, and orbitofrontal cortex. Ann N Y Acad Sci.

[37] Igarashi KM, Ieki N, An M, Yamaguchi Y, Nagayama S, Kobayakawa K (2012). Parallel mitral and tufted cell pathways route distinct odor information to different targets in the olfactory cortex. J Neurosci.

[38] Gire DH, Schoppa NE (2009). Control of on/off glomerular signaling by a local GABAergic microcircuit in the olfactory bulb. J Neurosci.

[39] Yamaguchi M, Manabe H, Murata K, Mori K. Reorganization of neuronal circuits of the central olfactory system during postprandial sleep. Front Neural Circuits [Internet]. 2013 [cited 2020 Jul 13];7. Available from: http://journal.frontiersin.org/article/10.3389/fncir.2013.00132/abstract 10.3389/fncir.2013.00132 PMC3743305 23966911

[40] Courtiol E, Wilson DA. The olfactory thalamus: unanswered questions about the role of the mediodorsal thalamic nucleus in olfaction. Front Neural Circuits [Internet]. 2015 [cited 2020 Jul 13];9. Available from: http://journal.frontiersin.org/Article/10.3389/fncir.2015.00049/abstract 10.3389/fncir.2015.00049 PMC4585119 26441548

[41] Schwalbe G. Die Arachnoidalraum, ein Lymphraum und sein Zusammenhang mit dem Perichorioidalraum. Zbl Med Wiss. 1869;465–467.

[42] Dubey SK, Lakshmi KK, Krishna KV, Agrawal M, Singhvi G, Saha RN (2020). Insulin mediated novel therapies for the treatment of Alzheimer’s disease. Life Sci.

[43] Maimaiti S, Anderson KL, DeMoll C, Brewer LD, Rauh BA, Gant JC (2016). Intranasal insulin improves age-related cognitive deficits and reverses electrophysiological correlates of brain aging. J Gerontol A Biol Sci Med Sci.

[44] Pang Y, Lin S, Wright C, Shen J, Carter K, Bhatt A (2016). Intranasal insulin protects against substantia nigra dopaminergic neuronal loss and alleviates motor deficits induced by 6-OHDA in rats. Neuroscience.

[45] Rabiee N, Ahmadi S, Afshari R, Khalaji S, Rabiee M, Bagherzadeh M, et al. Polymeric nanoparticles for nasal drug delivery to the brain: relevance to Alzheimer’s disease. Adv Ther. 2020;2000076.

[46] Wu H, Hu K, Jiang X (2008). From nose to brain: understanding transport capacity and transport rate of drugs. Expert Opin Drug Deliv.

[47] Erdő F, Bors LA, Farkas D, Bajza Á, Gizurarson S (2018). Evaluation of intranasal delivery route of drug administration for brain targeting. Brain Res Bull.

[48] Renner DB, Svitak AL, Gallus NJ, Ericson ME, Frey WH, Hanson LR (2012). Intranasal delivery of insulin via the olfactory nerve pathway: insulin delivery via olfactory nerve pathway. J Pharm Pharmacol.

[49] Gopinath P, Gopinath G, Kumar A. Target site of intranasally sprayed substances and their transport across the nasal mucosa: a new insight into the intranasal route of drug-delivery. Current Ther Res. 1978;596–607.

[50] De Lorenzo AJD. The olfactory neuron and the blood-brain barrier. In: Wolstenholme GEW, Knight J, editors. Novartis Found Symp [Internet]. Chichester, UK: John Wiley & Sons, Ltd.; 2008 [cited 2020 Jul 13]. p. 151–76. Available from: http://doi.wiley.com/10.1002/9780470715369.ch9

[51] Perl D, Good P (1987). Uptake of aluminium into central nervous system along nasal-olfactory pathways. The Lancet.

[52] Balin BJ, Broadwell RD, Salcman M, El-Kalliny M (1986). Avenues for entry of peripherally administered protein to the central nervous system in mouse, rat, and squirrel monkey. J Comp Neurol.

[53] Broadwell RD, Balin BJ (1985). Endocytic and exocytic pathways of the neuronal secretory process and trans synaptic transfer of wheat germ agglutinin-horseradish peroxidasein vivo. J Comp Neurol.

[54] Baker H, Spencer RF (1986). Transneuronal transport of peroxidase-conjugated wheat germ agglutinin (WGA-HRP) from the olfactory epithelium to the brain of the adult rat. Exp Brain Res.

[55] Shipley MT (1985). Transport of molecules from nose to brain: transneuronal anterograde and retrograde labeling in the rat olfactory system by wheat germ agglutinin-horseradish peroxidase applied to the nasal epithelium. Brain Res Bull.

[56] Thorne RG, Emory CR, Ala TA, Frey WH (1995). Quantitative analysis of the olfactory pathway for drug delivery to the brain. Brain Res.

[57] Deckner M-L, Frién J, Verge VMK, Hökfelt T, Risling M (1993). Localization of neurotrophin receptors in olfactory epithelium and bulb: NeuroReport.

[58] Baly C, Aioun J, Badonnel K, Lacroix M-C, Durieux D, Schlegel C (2007). Leptin and its receptors are present in the rat olfactory mucosa and modulated by the nutritional status. Brain Res.

[59] Caillol M, Aïoun J, Baly C, Persuy M-A, Salesse R. Localization of orexins and their receptors in the rat olfactory system: possible modulation of olfactory perception by a neuropeptide synthetized centrally or locally. Brain Res. 2003;960:48–61. 10.1016/s0006-8993(02)03755-1 12505657

[60] Hardy AB, Aïoun J, Baly C, Julliard KA, Caillol M, Salesse R (2005). Orexin A modulates mitral cell activity in the rat olfactory bulb: patch-clamp study on slices and immunocytochemical localization of orexin receptors. Endocrinology.

[61] Lacroix M-C, Rodriguez-Enfedaque A, Grébert D, Laziz I, Meunier N, Monnerie R (2011). Insulin but not leptin protects olfactory mucosa from apoptosis: insulin as an anti-apoptotic factor for olfactory mucosa. J Neuroendocrinol.

[62] Palouzier-Paulignan B, Lacroix M-C, Aime P, Baly C, Caillol M, Congar P (2012). Olfaction under metabolic influences. Chem Senses.

[63] Flexner S, Clark PF (1912). A note on the mode of infection in epidemic poliomyelitis. Exp Biol Med.

[64] Brodie M, Elvidge AR (1934). The portal of entry and transmission of the virus of poliomyelitis. Science.

[65] Schultz EW, Gebhardt LP (1936). Prevention of intranasally inoculated poliomyelitis in monkeys by previous intranasal irrigation with chemical agents. Exp Biol Med.

[66] Sulphate ZINC (1938). Spray for prevention of poliomyelitis. Br Med J.

[67] Koike S, Horie H, Ise I, Okitsu A, Yoshida M, Iizuka N (1990). The poliovirus receptor protein is produced both as membrane-bound and secreted forms. EMBO J.

[68] Racaniello VR (1996). Early events in poliovirus infection: virus-receptor interactions. Proc Natl Acad Sci.

[69] Aoki J, Koike S, Ise I, Sato-Yoshida Y, Nomoto A (1994). Amino acid residues on human poliovirus receptor involved in interaction with poliovirus. J Biol Chem.

[70] Connor RJ, Kawaoka Y, Webster RG, Paulson JC (1994). Receptor specificity in human, avian, and equine H2 and H3 influenza virus isolates. Virology.

[71] Shinya K, Ebina M, Yamada S, Ono M, Kasai N, Kawaoka Y (2006). Influenza virus receptors in the human airway. Nature.

[72] Kumlin U, Olofsson S, Dimock K, Arnberg N (2008). Sialic acid tissue distribution and influenza virus tropism. Influenza Other Respir Viruses.

[73] Milho R, Frederico B, Efstathiou S, Stevenson PG. A heparan-dependent herpesvirus targets the olfactory neuroepithelium for host entry. Coscoy L, editor. PLoS Pathog. 2012;8:e1002986. 10.1371/journal.ppat.1002986 PMC3486907 23133384

[74] Shivkumar M, Milho R, May JS, Nicoll MP, Efstathiou S, Stevenson PG (2013). Herpes simplex virus 1 targets the murine olfactory neuroepithelium for host entry. J Virol.

[75] Tan CSE, Stevenson PG (2014). B cell response to herpesvirus infection of the olfactory neuroepithelium. J Virol.

[76] Brann DH, Tsukahara T, Weinreb C, Lipovsek M, Van den Berge K, Gong B, et al. Non-neuronal expression of SARS-CoV-2 entry genes in the olfactory system suggests mechanisms underlying COVID-19-associated anosmia [Internet]. Neuroscience; 2020 Mar. Available from: http://biorxiv.org/lookup/10.1101/2020.03.25.009084 10.1126/sciadv.abc5801 PMC10715684 32937591

[77] Fodoulian L, Tuberosa J, Rossier D, Boillat M, Kan C, Pauli V, et al. SARS-CoV-2 receptor and entry genes are expressed by sustentacular cells in the human olfactory neuroepithelium [Internet]. Neuroscience; 2020 Apr. Available from: http://biorxiv.org/lookup/10.1101/2020.03.31.013268 10.1016/j.isci.2020.101839 PMC7685946 33251489

[78] Hoffmann M, Kleine-Weber H, Schroeder S, Krüger N, Herrler T, Erichsen S (2020). SARS-CoV-2 cell entry depends on ACE2 and TMPRSS2 and is blocked by a clinically proven protease inhibitor. Cell.

[79] Sungnak W, Bécavin C, Berg M, Queen R, Litvinukova M, Talavera-López C (2020). SARS-CoV-2 entry factors are highly expressed in nasal epithelial cells together with innate immune genes. Nat Med.

[80] Buchner K, Seitz-Tutter D, Schönitzer K, Weiss DG (1987). A quantitative study of anterograde and retrograde axonal transport of exogenous proteins in olfactory nerve C-fibers. Neuroscience.

[81] Lochhead JJ, Thorne RG (2012). Intranasal delivery of biologics to the central nervous system. Adv Drug Deliv Rev.

[82] Selvaraj K, Gowthamarajan K, Karri VVSR. Nose to brain transport pathways an overview: potential of nanostructured lipid carriers in nose to brain targeting. Artif Cells Nanomedicine Biotechnol. 2017;1–8. 10.1080/21691401.2017.1420073 29282995

[83] Robertson B, Grant G (1985). A comparison between wheat germ agglutinin- and choleragenoid-horseradish peroxidase as anterogradely transported markers in central branches of primary sensory neurones in the rat with some observations in the cat. Neuroscience.

[84] Anton F, Peppel P (1991). Central projections of trigeminal primary afferents innervating the nasal mucosa: a horseradish peroxidase study in the rat. Neuroscience.

[85] Hill JM, Ball MJ, Neumann DM, Azcuy AM, Bhattacharjee PS, Bouhanik S (2008). The high prevalence of herpes simplex virus type 1 DNA in human trigeminal ganglia is not a function of age or gender. J Virol.

[86] Ball MJ, Lukiw WJ, Kammerman EM, Hill JM (2013). Intracerebral propagation of Alzheimer’s disease: Strengthening evidence of a herpes simplex virus etiology. Alzheimers Dement.

[87] Steinke A, Meier-Stiegen S, Drenckhahn D, Asan E (2008). Molecular composition of tight and adherens junctions in the rat olfactory epithelium and fila. Histochem Cell Biol.

[88] Wolburg H, Wolburg-Buchholz K, Sam H, Horvát S, Deli MA, Mack AF (2008). Epithelial and endothelial barriers in the olfactory region of the nasal cavity of the rat. Histochem Cell Biol.

[89] Cowan CM, Roskams AJ (2002). Apoptosis in the mature and developing olfactory neuroepithelium. Microsc Res Tech.

[90] Doty RL, editor. Handbook of Olfaction and Gustation: Doty/Handbook of Olfaction and Gustation [Internet]. Hoboken, NJ, USA: John Wiley & Sons, Inc; 2015 [cited 2020 Jul 13]. Available from: http://doi.wiley.com/10.1002/9781118971758

[91] Liberia T, Martin-Lopez E, Meller SJ, Greer CA. Sequential maturation of olfactory sensory neurons in the mature olfactory epithelium. eneuro. 2019;6:ENEURO.0266–19.2019. 10.1523/ENEURO.0266-19.2019 PMC6795559 31554664

[92] Thorne RG, Pronk GJ, Padmanabhan V, Frey WH (2004). Delivery of insulin-like growth factor-I to the rat brain and spinal cord along olfactory and trigeminal pathways following intranasal administration. Neuroscience.

[93] van Velthoven CTJ, Kavelaars A, van Bel F, Heijnen CJ. Nasal administration of stem cells: a promising novel route to treat neonatal ischemic brain damage: Pediatr Res. 2010;1. 10.1203/PDR.0b013e3181f1c289 20639794

[94] Falcone JA, Salameh TS, Yi X, Cordy BJ, Mortell WG, Kabanov AV (2014). Intranasal administration as a route for drug delivery to the brain: evidence for a unique pathway for albumin. J Pharmacol Exp Ther.

[95] Galeano C, Qiu Z, Mishra A, Farnsworth SL, Hemmi JJ, Moreira A (2018). The route by which intranasally delivered stem cells enter the central nervous system. Cell Transplant.

[96] Lochhead JJ, Kellohen KL, Ronaldson PT, Davis TP. Distribution of insulin in trigeminal nerve and brain after intranasal administration. Sci Rep [Internet]. 2019 [cited 2020 Jul 13];9. Available from: http://www.nature.com/articles/s41598-019-39191-5 10.1038/s41598-019-39191-5 PMC6385374 30796294

[97] Deli MA (2009). Potential use of tight junction modulators to reversibly open membranous barriers and improve drug delivery. Biochim Biophys Acta BBA - Biomembr.

[98] Ghadiri M, Young P, Traini D (2019). Strategies to enhance drug absorption via nasal and pulmonary routes. Pharmaceutics.

[99] Nedelcovych MT, Gadiano AJ, Wu Y, Manning AA, Thomas AG, Khuder SS (2018). Pharmacokinetics of intranasal versus subcutaneous insulin in the mouse. ACS Chem Neurosci.

[100] Yoffey JM, Sullivan ER, Drinker CK (1938). The lymphatic pathway from the nose and pharynx. J Exp Med.

[101] Yang W, Jin B-H, Chen Y-J, Cao C, Zhu J-Z, Zhao Y-Z (2019). The involvement of perivascular spaces or tissues in the facial intradermal brain-targeted delivery. Drug Deliv.

[102] Hadaczek P, Yamashita Y, Mirek H, Tamas L, Bohn MC, Noble C (2006). The “perivascular pump” driven by arterial pulsation is a powerful mechanism for the distribution of therapeutic molecules within the brain. Mol Ther.

[103] Djupesland PG, Mahmoud RA, Messina JC (2013). Accessing the brain: the nose may know the way. J Cereb Blood Flow Metab.

[104] Lochhead JJ, Wolak DJ, Pizzo ME, Thorne RG (2015). Rapid transport within cerebral perivascular spaces underlies widespread tracer distribution in the brain after intranasal administration. J Cereb Blood Flow Metab.

[105] Fortuna A, Alves G, Serralheiro A, Sousa J, Falcão A (2014). Intranasal delivery of systemic-acting drugs: Small-molecules and biomacromolecules. Eur J Pharm Biopharm.

[106] Schwarz B, Merkel OM (2019). Nose-to-brain delivery of biologics Ther Deliv.

[107] Grassin-Delyle S, Buenestado A, Naline E, Faisy C, Blouquit-Laye S, Couderc L-J (2012). Intranasal drug delivery: an efficient and non-invasive route for systemic administration. Pharmacol Ther.

[108] Agrawal M, Saraf S, Saraf S, Antimisiaris SG, Hamano N, Li S-D (2018). Recent advancements in the field of nanotechnology for the delivery of anti-Alzheimer drug in the brain region. Expert Opin Drug Deliv.

[109] Illum L (2000). Transport of drugs from the nasal cavity to the central nervous system. Eur J Pharm Sci.

[110] Djupesland PG (2013). Nasal drug delivery devices: characteristics and performance in a clinical perspective—a review. Drug Deliv Transl Res.

[111] Kublik H, Vidgren M (1998). Nasal delivery systems and their effect on deposition and absorption. Adv Drug Deliv Rev.

[112] Hussein NR. Chapter 15 - Advances in nasal drug delivery systems. 33.

[113] Salib RJ, Howarth PH (2003). Safety and tolerability profiles of intranasal antihistamines and intranasal corticosteroids in the treatment of allergic rhinitis. Drug Saf..

[114] Costantino HR, Illum L, Brandt G, Johnson PH, Quay SC (2007). Intranasal delivery: physicochemical and therapeutic aspects. Int J Pharm.

[115] Janssens MM-L, Vanden Bussche G. Levocabastine: an effective topical treatment of allergic rhinoconjunctivitis. Clin Htmlent Glyphamp Asciiamp Exp Allergy. 1991;21:29–36. 10.1111/j.1365-2222.1991.tb01755.x 1680536

[116] Hampel FC, Martin BG, Dolen J, Travers S, Karcher K, Holton D (1999). Efficacy and safety of levocabastine nasal spray for seasonal allergic rhinitis. Am J Rhinol.

[117] Barnes PJ (1997). Molecular mechanisms of glucocorticoid action in asthma. Pulm Pharmacol Ther.

[118] Behl C, Pimplaskar H, Sileno A, deMeireles J, Romeo V (1998). Effects of physicochemical properties and other factors on systemic nasal drug delivery. Adv Drug Deliv Rev.

[119] Battaglia L, Panciani PP, Muntoni E, Capucchio MT, Biasibetti E, De Bonis P (2018). Lipid nanoparticles for intranasal administration: application to nose-to-brain delivery. Expert Opin Drug Deliv.

[120] Li BV, Jin F, Lee SL, Bai T, Chowdhury B, Caramenico HT (2013). Bioequivalence for locally acting nasal spray and nasal aerosol products: standard development and generic approval. AAPS J.

[121] Landau AJ, Frishman WH, Alturk N, Adjei-Poku M, Fornasier-Bongo M, Furia S (1993). Improvement in exercise tolerance and immediate beta-adrenergic blockade with intranasal propranolol in patients with angina pectoris. Am J Cardiol.

[122] Landau AJ, Eberhardt RT, Frishman WH (1994). Intranasal delivery of cardiovascular agents: an innovative approach to cardiovascular pharmacotherapy. Am Heart J.

[123] Illum L, Watts P, Fisher AN, Hinchcliffe M, Norbury H, Jabbal-Gill I (2002). Intranasal delivery of morphine. J Pharmacol Exp Ther.

[124] Fitzgibbon D, Morgan D, Dockter D, Barry C, Kharasch ED (2003). Initial pharmacokinetic, safety and efficacy evaluation of nasal morphine gluconate for breakthrough pain in cancer patients. Pain..

[125] Illum L (2012). Nasal drug delivery — recent developments and future prospects. J Controlled Release.

[126] EMA C. Assessment report Nyxoid International non-proprietary name: naloxone. EMA/CHMP/690823/2017.

[127] Björkman S, Rigemar G, Idvall J (1997). Pharmacokinetics of midazolam given as an intranasal spray to adult surgical patients. Br J Anaesth.

[128] Wermeling DP (2009). Intranasal delivery of antiepileptic medications for treatment of seizures. Neurotherapeutics.

[129] Wilson MT (2004). Nasal/buccal midazolam use in the community. Arch Dis Child.

[130] Fişgin T, Gurer Y, Tezic T, Senbil N, Zorlu P, Okuyaz C (2002). Effects of intranasal midazolam and rectal diazepam on acute convulsions in children: prospective randomized study. J Child Neurol.

[131] Scott RT, Ross B, Anderson C, Archer DF (1991). Pharmacokinetics of percutaneous estradiol: a crossover study using a gel and a transdermal system in comparison with oral micronized estradiol. Obstet Gynecol.

[132] Studd J, Pornel B, Marton I, Bringer J, Varin C, Tsouderos Y (1999). Efficacy and acceptability of intranasal 17 β-oestradiol for menopausal symptoms: randomised dose-response study. The Lancet.

[133] Gompel A, Bergeron C, Jondet M, Dhont M, Van der Mooren MJ, Toth KS (2000). Endometrial safety and tolerability of AERODIOL® (intranasal estradiol) for 1 year. Maturitas.

[134] Mattsson LA, Christiansen C, Colau J-C, Palacios S, Kenemans P, Bergeron C (2000). Clinical equivalence of intranasal and oral 17β-estradiol for postmenopausal symptoms. Am J Obstet Gynecol.

[135] Robinson AG (1976). DDAVP in the treatment of central diabetes insipidus. N Engl J Med.

[136] Waxman JH, Wass JAH, Hendry WF, Whitfield HN, Bary P, Besser GM (1983). Treatment of advanced prostatic cancer with buserelin, an analogue of gonadotrophin releasing hormone. Br J Urol.

[137] Franco JG, Baruffi RL, Mauri AL, Petersen CG, Chufallo JE, Felipe V (2001). Prospective randomized comparison of ovarian blockade with nafarelin versus leuprolide during ovarian stimulation with recombinant FSH in an ICSI program. J Assist Reprod Genet.

[138] Morimoto K, Katsumata H, Yabuta T, Iwanaga K, Kakemi M, Tabata Y (2001). Evaluation of gelatin microspheres for nasal and intramuscular administrations of salmon calcitonin. Eur J Pharm Sci.

[139] Gill JC, Ottum M, Schwartz B (2002). Evaluation of high concentration intranasal and intravenous desmopressin in pediatric patients with mild hemophilia A or mild-to-moderate type 1 von Willebrand disease. J Pediatr.

[140] Singh AK, Singh A, Madhv N. S. Nasal cavity, a promising transmucosal platform for drug delivery and research approaches from nasal to brain targetting. J Drug Deliv Ther [Internet]. 2012 [cited 2020 Jul 13];2. Available from: http://jddtonline.info/index.php/jddt/article/view/163

[141] Pardridge WM (2005). The blood-brain barrier: bottleneck in brain drug development. NeuroRX.

[142] de Boer AG, Gaillard PJ (2007). Drug targeting to the brain. Annu Rev Pharmacol Toxicol.

[143] Saraiva C, Praça C, Ferreira R, Santos T, Ferreira L, Bernardino L (2016). Nanoparticle-mediated brain drug delivery: overcoming blood–brain barrier to treat neurodegenerative diseases. J Controlled Release.

[144] Cordon-Cardo C, O’Brien JP, Casals D, Rittman-Grauer L, Biedler JL, Melamed MR (1989). Multidrug-resistance gene (P-glycoprotein) is expressed by endothelial cells at blood-brain barrier sites. Proc Natl Acad Sci.

[145] Graff CL, Pollack GM (2005). Functional evidence for P-glycoprotein at the nose-brain barrier. Pharm Res.

[146] Khan AR, Liu M, Khan MW, Zhai G (2017). Progress in brain targeting drug delivery system by nasal route. J Controlled Release.

[147] Nicholson C (2001). Diffusion and related transport mechanisms in brain tissue. Rep Prog Phys.

[148] Wolak DJ, Thorne RG (2013). Diffusion of macromolecules in the brain: implications for drug delivery. Mol Pharm.

[149] Tønnesen J, Inavalli VVGK, Nägerl UV (2018). Super-resolution imaging of the extracellular space in living brain tissue. Cell.

[150] Helmbrecht H, Joseph A, McKenna M, Zhang M, Nance E (2020). Governing transport principles for nanotherapeutic application in the brain. Curr Opin Chem Eng.

[151] Seo Y-E, Bu T, Saltzman WM (2017). Nanomaterials for convection-enhanced delivery of agents to treat brain tumors. Curr Opin Biomed Eng.

[152] Li J, Zhao J, Tan T, Liu M, Zeng Z, Zeng Y (2020). Nanoparticle drug delivery system for glioma and its efficacy improvement strategies: a comprehensive review. Int J Nanomedicine.

[153] Bruinsmann FA, Richter Vaz G, de Cristo Soares Alves A, Aguirre T, Raffin Pohlmann A, Stanisçuaski Guterres S, et al. Nasal drug delivery of anticancer drugs for the treatment of glioblastoma: preclinical and clinical trials. Molecules. 2019;24:4312. 10.3390/molecules24234312 PMC6930669 31779126

[154] Peterson A, Bansal A, Hofman F, Chen TC, Zada G (2014). A systematic review of inhaled intranasal therapy for central nervous system neoplasms: an emerging therapeutic option. J Neurooncol.

[155] Hashizume R, Ozawa T, Gryaznov SM, Bollen AW, Lamborn KR, Frey WH (2008). New therapeutic approach for brain tumors: intranasal delivery of telomerase inhibitor GRN163. Neuro-Oncol.

[156] Thorne RG, Hanson LR, Ross TM, Tung D, Frey WH (2008). Delivery of interferon-β to the monkey nervous system following intranasal administration. Neuroscience.

[157] Shingaki T, Inoue D, Furubayashi T, Sakane T, Katsumi H, Yamamoto A (2010). Transnasal delivery of methotrexate to brain tumors in rats: a new strategy for brain tumor chemotherapy. Mol Pharm.

[158] Fonseca COD, Teixeira RM, Ramina R, Kovaleski G, Silva JT, Nagel J (2011). Case of advanced recurrent glioblastoma successfully treated with monoterpene perillyl alcohol by intranasal administration. J Cancer Ther.

[159] Santos J, Da Cruz WM, Schï¿½nthal A, Salazar M, Fontes CA, Quirico‑Santos T, et al. Efficacy of a ketogenic diet with concomitant intranasal perillyl alcohol as a novel strategy for the therapy of recurrent glioblastoma. Oncol Lett [Internet]. 2017 [cited 2020 Jul 13]; Available from: http://www.spandidos-publications.com/10.3892/ol.2017.7362 10.3892/ol.2017.7362 PMC5769394 29391903

[160] Chen T, da Fonseca C, Schönthal A (2018). Intranasal perillyl alcohol for glioma therapy: molecular mechanisms and clinical development. Int J Mol Sci.

[161] Kosfeld M, Heinrichs M, Zak PJ, Fischbacher U, Fehr E (2005). Oxytocin increases trust in humans. Nature.

[162] Guastella AJ, Einfeld SL, Gray KM, Rinehart NJ, Tonge BJ, Lambert TJ (2010). Intranasal oxytocin improves emotion recognition for youth with autism spectrum disorders. Biol Psychiatry.

[163] Quintana DS, Smerud KT, Andreassen OA, Djupesland PG (2018). Evidence for intranasal oxytocin delivery to the brain: recent advances and future perspectives. Ther Deliv.

[164] Deadwyler SA, Porrino L, Siegel JM, Hampson RE (2007). Systemic and nasal delivery of orexin-A (hypocretin-1) reduces the effects of sleep deprivation on cognitive performance in nonhuman primates. J Neurosci.

[165] Baier PC, Weinhold SL, Huth V, Gottwald B, Ferstl R, Hinze-Selch D (2008). Olfactory dysfunction in patients with narcolepsy with cataplexy is restored by intranasal orexin A (hypocretin-1). Brain.

[166] Dhuria SV, Hanson LR, Frey WH (2010). Intranasal delivery to the central nervous system: mechanisms and experimental considerations. J Pharm Sci.

[167] Fliedner S, Schulz C, Lehnert H (2006). Brain uptake of intranasally applied radioiodinated leptin in Wistar rats. Endocrinology.

[168] Schulz C, Paulus K, Jöhren O, Lehnert H (2012). Intranasal leptin reduces appetite and induces weight loss in rats with diet-induced obesity (DIO). Endocrinology.

[169] Berger S, Pho H, Fleury-Curado T, Bevans-Fonti S, Younas H, Shin M-K (2019). Intranasal leptin relieves sleep-disordered breathing in mice with diet-induced obesity. Am J Respir Crit Care Med.

[170] Stockhorst U, de Fries D, Steingrueber H-J, Scherbaum WA (2004). Insulin and the CNS: effects on food intake, memory, and endocrine parameters and the role of intranasal insulin administration in humans. Physiol Behav.

[171] Banks WA, Owen JB, Erickson MA (2012). Insulin in the brain: there and back again. Pharmacol Ther.

[172] Craft S, Peskind E, Schwartz MW, Schellenberg GD, Raskind M, Porte D (1998). Cerebrospinal fluid and plasma insulin levels in Alzheimer’s disease: relationship to severity of dementia and apolipoprotein E genotype. Neurology.

[173] Hoyer S (2002). The brain insulin signal transduction system and sporadic (type II) Alzheimer disease: an update. J Neural Transm.

[174] Kianpour Rad S, Arya A, Karimian H, Madhavan P, Rizwan F, Koshy S (2018). Mechanism involved in insulin resistance via accumulation of &beta;-amyloid and neurofibrillary tangles: link between type 2 diabetes and Alzheimer&rsquo;s disease. Drug Des Devel Ther.

[175] Reger MA, Watson GS, Frey WH, Baker LD, Cholerton B, Keeling ML (2006). Effects of intranasal insulin on cognition in memory-impaired older adults: modulation by APOE genotype. Neurobiol Aging.

[176] Reger MA, Watson GS, Green PS, Baker LD, Cholerton B, Fishel MA (2008). Intranasal insulin administration dose-dependently modulates verbal memory and plasma amyloid-beta in memory-impaired older adults. J Alzheimers Dis JAD.

[177] Claxton A, Baker LD, Hanson A, Trittschuh EH, Cholerton B, Morgan A (2015). Long-acting intranasal insulin detemir improves cognition for adults with mild cognitive impairment or early-stage Alzheimer’s disease dementia. J Alzheimers Dis.

[178] Craft S, Claxton A, Baker LD, Hanson A, Cholerton B, Trittschuh EH, et al. Effects of regular and long-acting insulin on cognition and Alzheimer’s disease biomarkers: a pilot clinical trial. de la Monte S, editor. J Alzheimers Dis. 2017;57:1325–34. 10.3233/JAD-161256 PMC5409050 28372335

[179] Malhotra M, Tomaro-Duchesneau C, Saha S, Prakash S (2013). Intranasal, siRNA delivery to the brain by TAT/MGF tagged PEGylated chitosan nanoparticles. J Pharm.

[180] Rodriguez M, Lapierre J, Ojha CR, Kaushik A, Batrakova E, Kashanchi F, et al. Intranasal drug delivery of small interfering RNA targeting Beclin1 encapsulated with polyethylenimine (PEI) in mouse brain to achieve HIV attenuation. Sci Rep [Internet]. 2017 [cited 2020 Jul 14];7. Available from: http://www.nature.com/articles/s41598-017-01819-9 10.1038/s41598-017-01819-9 PMC5431946 28500326

[181] Alarcón-Arís D, Recasens A, Galofré M, Carballo-Carbajal I, Zacchi N, Ruiz-Bronchal E (2018). Selective α-synuclein knockdown in monoamine neurons by intranasal oligonucleotide delivery: potential therapy for Parkinson’s disease. Mol Ther.

[182] Scoles DR, Minikel EV, Pulst SM (2019). Antisense oligonucleotides: a primer Neurol Genet.

[183] Danielyan L, Schäfer R, von Ameln-Mayerhofer A, Buadze M, Geisler J, Klopfer T (2009). Intranasal delivery of cells to the brain. Eur J Cell Biol.

[184] Reitz M, Demestre M, Sedlacik J, Meissner H, Fiehler J, Kim SU (2012). Intranasal delivery of neural stem/progenitor cells: a noninvasive passage to target intracerebral glioma. STEM CELLS Transl Med.

[185] van Woensel M, Wauthoz N, Rosière R, Amighi K, Mathieu V, Lefranc F (2013). Formulations for Intranasal delivery of pharmacological agents to combat brain disease: a new opportunity to tackle GBM?. Cancers.

[186] Balyasnikova IV, Prasol MS, Ferguson SD, Han Y, Ahmed AU, Gutova M (2014). Intranasal Delivery of mesenchymal stem cells significantly extends survival of irradiated mice with experimental brain tumors. Mol Ther.

[187] Li G, Bonamici N, Dey M, Lesniak MS, Balyasnikova IV (2018). Intranasal delivery of stem cell-based therapies for the treatment of brain malignancies. Expert Opin Drug Deliv.

[188] Yu-Taeger L, Stricker-Shaver J, Arnold K, Bambynek-Dziuk P, Novati A, Singer E (2019). Intranasal administration of mesenchymal stem cells ameliorates the abnormal dopamine transmission system and inflammatory reaction in the R6/2 mouse model of Huntington disease. Cells.

[189] Guo Y, Laube B, Dalby R (2005). The effect of formulation variables and breathing patterns on the site of nasal deposition in an anatomically correct model. Pharm Res.

[190] Scherließ R (2020). Nasal formulations for drug administration and characterization of nasal preparations in drug delivery. Ther Deliv.

[191] Kumar A, Pandey AN, Jain SK (2016). Nasal-nanotechnology: revolution for efficient therapeutics delivery. Drug Deliv.

[192] Quadir M, Zia H, Needham TE (1999). Toxicological implications of nasal formulations. Drug Deliv.

[193] Ugwoke M, Agu R, Verbeke N, Kinget R (2005). Nasal mucoadhesive drug delivery: background, applications, trends and future perspectives. Adv Drug Deliv Rev.

[194] Jiao J, Zhang L (2019). Influence of intranasal drugs on human nasal mucociliary clearance and ciliary beat frequency. Allergy Asthma Immunol Res.

[195] Bende M, Hansell P, Intaglietta M, Arfors K-E (1992). Effect of oxymetazoline nose drops on vascular permeability of the nasal mucosa in the rabbit after provocation with leukotriene B_4_. ORL.

[196] Åkerlund A, Arfors K-E, Bende M, Intaglietta M (1993). Effect of oxymetazoline on nasal and sinus mucosal blood flow in the rabbit as measured with laser-Doppler flowmetry. Ann Otol Rhinol Laryngol.

[197] Loewen AHS (2004). Thunderclap headache and reversible segmental cerebral vasoconstriction associated with use of oxymetazoline nasal spray. Can Med Assoc J.

[198] Juniper E, Guyatt G, Obyrne P, Viveiros M (1990). Aqueous beclomethasone diproprionate nasal spray: regular versus “as required” use in the treatment of seasonal allergic rhinitis. J Allergy Clin Immunol.

[199] Stanaland B (2004). Once-daily budesonide aqueous nasal spray for allergic rhinitis: a review. Clin Ther.

[200] Thornton-Manning J, Dahl A (1997). Metabolic capacity of nasal tissue. Mutat Res Mol Mech Mutagen.

[201] Heydel J-M, Coelho A, Thiebaud N, Legendre A, Bon A-ML, Faure P, et al. Odorant- binding proteins and xenobiotic metabolizing enzymes: implications in olfactory perireceptor events: odorant-binding proteins and metabolizing enzymes. Anat Rec. 2013;296:1333–45. 10.1002/ar.22735 23907783

[202] Dale O, Hjortkjaer R, Kharasch ED (2002). Nasal administration of opioids for pain management in adults. Acta Anaesthesiol Scand.

[203] Washington N, Steele RJ, Jackson S, Bush D, Mason J, Gill D (2000). Determination of baseline human nasal pH and the effect of intranasally administered buffers. Int J Pharm.

[204] Marttin E, Schipper NG, Verhoef JC, Merkus FWH (1998). Nasal mucociliary clearance as a factor in nasal drug delivery. Adv Drug Deliv Rev.

[205] Mickenhagen A, Siefer O, Neugebauer P, Stennert E (2008). Der Einfluss verschiedener α-Sympathomimetika und Benzalkoniumchlorid auf die Zilienschlagfrequenz humaner Flimmerzellen in vitro. Laryngo-Rhino-Otol.

[206] Zhang L, Han D, Song X, Wang K, Wang H (2008). Effect of oxymetazoline on healthy human nasal ciliary beat frequency measured with high-speed digital microscopy and mucociliary transport time. Ann Otol Rhinol Laryngol.

[207] Stanley PJ, Griffin WM, Wilson R, Greenstone MA, Mackay IS, Cole PJ (1985). Effect of betamethasone and betamethasone with neomycin nasal drops on human nasal mucociliary clearance and ciliary beat frequency. Thorax.

[208] Alberty J, Stoll W (1998). The effect of antiallergic intranasal formulations on ciliary beat frequency of human nasal epithelium in vitro. Allergy.

[209] Koidl B, Hofmann T, Wolf G (1998). Die Wirkung topischer Kortikosteroide und topischer Antihistaminika auf das Flimmerepithel humaner Nasenschleimhaut in vitro. HNO.

[210] van de Donk HJ, Zuidema J, Merkus FW (1981). The effects of nasal drops on the ciliary beat frequency of chicken embryo tracheas. Rhinology.

[211] Joki S, Saano V, Nuutinen J, Virta P, Karttunen P, Silvasti M (1996). Effects of some preservative agents on rat and guinea pig tracheal and human nasal ciliary beat frequency. Am J Rhinol.

[212] Kuboyama Y, Suzuki K, Hara T (1997). Nasal lesions induced by intranasal administration of benzalkonium chloride in rats. J Toxicol Sci.

[213] Bernstein IL (2000). Is the use of benzalkonium chloride as a preservative for nasal formulations a safety concern? A cautionary note based on compromised mucociliary transport. J Allergy Clin Immunol.

[214] Berg ØH, Lie K, Steinsvåg SK (1997). The effects of topical nasal steroids on rat respiratory mucosa in vivo, with special reference to benzalkonium chloride. Allergy.

[215] Steinsvag SK, Bjerknes R, Berg ØH (1996). Effects of topical nasal steroids on human respiratory mucosa and human granulocytes in vitro. Acta Otolaryngol (Stockh).

[216] Davis SS, Illum L (2003). Absorption enhancers for nasal drug delivery: Clin Pharmacokinet.

[217] EMA. Benzalkonium chloride used as an excipient [Internet]. EMA/CHMP/495737/2013 Oct 9, 2017 p. 14. Available from: https://www.ema.europa.eu/en/documents/report/benzalkonium-chloride-used-excipient-report-published-support-questions-answers-benzalkonium_en.pdf

[218] Lopes P, Bruschi F, Foidart J-M, Calaf J (2000). Randomized comparison of intranasal and transdermal estradiol.

[219] Mula M (2014). The safety and tolerability of intranasal midazolam in epilepsy. Expert Rev Neurother.

[220] Thakker A, Shanbag P (2013). A randomized controlled trial of intranasal-midazolam versus intravenous-diazepam for acute childhood seizures. J Neurol.

[221] Hogan RE, Gidal BE, Koplowitz B, Koplowitz LP, Lowenthal RE, Carrazana E (2020). Bioavailability and safety of diazepam intranasal solution compared to oral and rectal diazepam in healthy volunteers. Epilepsia.

[222] Sheng J, Liu S, Qin H, Li B, Zhang X. Drug-resistant epilepsy and surgery. Curr Neuropharmacol [Internet]. 2017 [cited 2020 Nov 27];16. Available from: http://www.eurekaselect.com/152157/article 10.2174/1570159X15666170504123316 PMC5771378 28474565

[223] Shringarpure M, Gharat S, Momin M, Omri A. Management of epileptic disorders using nanotechnology-based strategies for nose-to-brain drug delivery. Expert Opin Drug Deliv. 2020;1–17. 10.1080/17425247.2021.1823965 32921169

[224] El-Enin HA, AL-Shanbari AH. Nanostructured liquid crystalline formulation as a remarkable new drug delivery system of anti-epileptic drugs for treating children patients. Saudi Pharm J. 2018;26:790–800. 10.1016/j.jsps.2018.04.004 PMC6128721 30202219

[225] Naqvi S, Panghal A, Flora SJS. Nanotechnology: a promising approach for delivery of neuroprotective drugS. Front Neurosci [Internet]. 2020 [cited 2020 Nov 27];14. Available from: https://www.frontiersin.org/article/10.3389/fnins.2020.00494/full 10.3389/fnins.2020.00494 PMC7297271 32581676

[226] Tan MSA, Parekh HS, Pandey P, Siskind DJ, Falconer JR (2020). Nose-to-brain delivery of antipsychotics using nanotechnology: a review. Expert Opin Drug Deliv.

[227] Ansari MA, Chung I-M, Rajakumar G, Alzohairy MA, Alomary MN, Thiruvengadam M (2020). Current nanoparticle approaches in nose to brain drug delivery and anticancer therapy - a review. Curr Pharm Des.

[228] Dokuyucu R, Gokce H, Sahan M, Sefil F, Tas ZA, Tutuk O (2015). Systemic side effects of locally used oxymetazoline. Int J Clin Exp Med.

[229] Nordt SP, Vivero LE, Cantrell FL (2016). Not just a drop in the bucket—inversion of oxymetazoline nasal decongestant container increases potential for severe pediatric poisoning. J Pediatr.

[230] Eddy O, Howell JM (2003). Are one or two dangerous? Clonidine and topical imidazolines exposure in toddlers. J Emerg Med.

[231] Latham GJ, Jardine DS (2013). Oxymetazoline and hypertensive crisis in a child: can we prevent it? Polaner D, editor. Pediatr Anesth.

[232] Tobias JD, Cartabuke R, Taghon T. Oxymetazoline (Afrin ^®^ ): maybe there is more that we need to know. Morton N, editor. Pediatr Anesth. 2014;24:795–8. 10.1111/pan.12399 25039870

[233] Rey E, Tréluyer J-M, Pons G (1999). Pharmacokinetic optimisation of benzodiazepine therapy for acute seizures: focus on delivery routes. Clin Pharmacokinet.

[234] Holsti M, Dudley N, Schunk J, Adelgais K, Greenberg R, Olsen C, et al. Intranasal midazolam vs rectal diazepam for the home treatment of acute seizures in pediatric patients with epilepsy. Arch Pediatr Adolesc Med [Internet]. 2010 [cited 2020 Jul 13];164. Available from: http://archpedi.jamanetwork.com/article.aspx?10.1001/archpediatrics.2010.130 10.1001/archpediatrics.2010.130 20679166

[235] Heytens L, Camu F (1984). Pulmonary edema during cesarean section related to the use of oxytocic drugs. Acta Anaesthesiol Belg.

[236] Roberts NV, Keast PJ, Brodeky V, Oates A, Ritchie BC (1992). The effects of oxytocin on the pulmonary and systemic circulation in pregnant ewes. Anaesth Intensive Care.

[237] Ghai B, Vayjnath AM, Lal S (2006). Acute pulmonary oedema following oxytocin administration: a life threatening complication. J Indian Med Assoc.

[238] Dogdu O, Yarlioglues M, Inanc T, Ardic I, Zencir C, Kaya MG (2011). Fatal pulmonary oedema following oxytocin administration in a pregnant woman with acute myocardial infarction. Cardiovasc Toxicol.

[239] Ohlsson B, Truedsson M, Bengtsson M, Torstenson R, Sjolund K, Bjornsson ES (2005). Effects of long-term treatment with oxytocin in chronic constipation; a double blind, placebo-controlled pilot trial. Neurogastroenterol Motil.

[240] Martins DA, Mazibuko N, Zelaya F, Vasilakopoulou S, Loveridge J, Oates A, et al. Effects of route of administration on oxytocin-induced changes in regional cerebral blood flow in humans. Nat Commun [Internet]. 2020 [cited 2020 Jul 13];11. Available from: http://www.nature.com/articles/s41467-020-14845-5 10.1038/s41467-020-14845-5 PMC7054359 32127545

[241] Anagnostou E, Soorya L, Chaplin W, Bartz J, Halpern D, Wasserman S (2012). Intranasal oxytocin versus placebo in the treatment of adults with autism spectrum disorders: a randomized controlled trial. Mol Autism.

[242] DeMayo MM, Song YJC, Hickie IB, Guastella AJ (2017). A review of the safety, efficacy and mechanisms of delivery of nasal oxytocin in children: therapeutic potential for autism and prader-willi syndrome, and recommendations for future research. Pediatr Drugs.

[243] Mitri J, Pittas AG (2009). Inhaled insulin—what went wrong. Nat Clin Pract Endocrinol Metab.

[244] Ceglia L, Lau J, Pittas AG (2006). Meta-analysis: efficacy and safety of inhaled insulin therapy in adults with diabetes mellitus. Ann Intern Med.

[245] Kling J (2008). Inhaled insulin’s last gasp?. Nat Biotechnol.

[246] Shapiro H, Kagan I, Shalita-Chesner M, Singer J, Singer P (2010). Inhaled aerosolized insulin: a “topical” anti-inflammatory treatment for acute lung injury and respiratory distress syndrome?. Inflammation.

[247] Heinemann L, Parkin CG (2018). Rethinking the viability and utility of inhaled insulin in clinical practice. J Diabetes Res.

[248] Choi H-Y, Lee Y-H, Lim C-H, Kim Y-S, Lee I-S, Jo J-M, et al. Assessment of respiratory and systemic toxicity of benzalkonium chloride following a 14-day inhalation study in rats. Part Fibre Toxicol [Internet]. 2020 [cited 2020 Jul 13];17. Available from: https://particleandfibretoxicology.biomedcentral.com/articles/10.1186/s12989-020-0339-8 10.1186/s12989-020-0339-8 PMC6986023 31992310

[249] Xue Y, Hieda Y, Saito Y, Nomura T, Fujihara J, Takayama K (2004). Distribution and disposition of benzalkonium chloride following various routes of administration in rats. Toxicol Lett.

[250] Code of Federal Regulations [Internet]. 2019. Available from: https://ecfr.federalregister.gov/current/title-21/chapter-I/subchapter-D/part-314/subpart-B/section-314.54

[251] FDA/ CDER. Draft Guidance for Industry, Applications Covered by Section 505(b)(2) [Internet]. 1999. Available from: https://www.fda.gov/media/72419/download

[252] FDA/ CDER. Determining whether to submit an ANDA or a 505(b)(2) application guidance for industry [Internet]. FDA-2017-D-5974 2019. Available from: https://www.fda.gov/media/124848/download

[253] Lyapustina S (2018). Regulatory pitfalls and opportunities when repurposing for inhalation therapy. Adv Drug Deliv Rev.

[254] FDA/ CDER. Nonclinical safety evaluation of reformulated drug products and products intended for administration by an alternate route guidance for industry and review staff. FDA-2008-D-0142 2015.

[255] Salminen WF, Wiles ME, Stevens RE (2019). Streamlining nonclinical drug development using the FDA 505(b)(2) new drug application regulatory pathway. Drug Discov Today.

[256] FDA. Human factors studies and related clinical study considerations in combination product design and development draft guidance for industry and FDA staff [Internet]. 2016. Available from: https://www.fda.gov/media/96018/download

[257] FDA/ CDER. Comparative analyses and related comparative use human factors studies for a drug-device combination product submitted in an ANDA: draft guidance for industry [Internet]. FDA-2016-D-4412 2017. Available from: https://www.fda.gov/media/102349/download

[258] FDA/ CDRH. Applying human factors and usability engineering to medical devices [Internet]. FDA-2011-D-0469 2016. Available from: https://www.fda.gov/media/80481/download

[259] FDA/ CDER. Guidance for industry nasal spray and inhalation solution, suspension, and spray drug products — chemistry, manufacturing, and controls documentation [Internet]. FDA-1999-D-0060 2002. Available from: https://www.fda.gov/regulatory-information/search-fda-guidance-documents/nasal-spray-and-inhalation-solution-suspension-and-spray-drug-products-chemistry-manufacturing-and

[260] FDA/ CDER. Metered dose inhaler (MDI) and dry powder inhaler (DPI) products - quality considerations guidance for industry. FDA-2018-D-1098 2018.

